# Astrocyte Proximity Protects Synapses From Human Amyloid‐Beta Induced Degeneration in a Mouse Ex Vivo Model of Early Alzheimer's Disease

**DOI:** 10.1111/ejn.70480

**Published:** 2026-03-28

**Authors:** Francesco Gobbo, Declan King, Jane Tulloch, Davide Gobbo, Calum Bonthron, Soraya Meftah, Caleb Stoddart‐Campbelton, Arisa Tamura, Jamie Rose, Colin Smith, Claire Durrant, Tara L. Spires‐Jones

**Affiliations:** ^1^ Institute for Neuroscience and Cardiovascular Research and UK Dementia Research Institute University of Edinburgh Edinburgh UK; ^2^ Department of Molecular Physiology, Centre for Integrative Physiology and Molecular Medicine (CIPMM) University of Saarland Homburg Germany; ^3^ Department of Biological Sciences Tokyo Metropolitan University Tokyo Japan

**Keywords:** Alzheimer's disease, astrocytes, Aβ, synapse, synapse loss

## Abstract

Synapse loss is the strongest pathological correlate of cognitive decline in Alzheimer's disease (ad) and is most pronounced around amyloid plaque pathology in the brain. Although mechanisms remain incompletely understood, hyperactivity downstream of soluble amyloid beta (Aβ) is strongly implicated in synapse degeneration. Engulfment of synapses by reactive astrocytes was observed in end‐stage disease tissue, particularly around plaques. Due to astrocytes' role in synaptic modulation, we hypothesised that astrocytes could modulate synapse degeneration downstream of soluble Aβ earlier in disease pathogenesis. To test this, we challenged organotypic mouse brain slices with human ad brain homogenates containing Aβ. Changes in synaptic activity were detected 2 h after Aβ challenge, and spine loss was seen after 24 h. We observe that Aβ‐containing homogenate induces a significant loss of spines compared with controls. Aβ‐containing homogenate also causes a significant increase in the frequency of synaptic calcium events, particularly in synapses lost at 24 h. Dendritic spines associated with astrocytic processes were significantly more likely to survive at 24 h after Aβ challenge and had reduced levels of externalised phosphatidyl serine despite no effect of astrocyte proximity on synaptic activity. Inhibiting astrocytic glutamate transporters prevented the protective effects of astrocytes on synapses, indicating that astrocytes are protective of synapses at least in part through removing excess glutamate from the synaptic microenvironment. Our findings suggest that an organotypic mouse brain slice model challenged with disease tissue homogenates effectively recapitulates key features of early AD, including synapse loss and hyperexcitability. Moreover, they indicate that astrocytes play a protective role in preserving synapses, particularly during short‐term exposure to low concentrations of toxic Aβ. Future work is needed to elucidate the role of astrocyte‐mediated synapse phagocytosis in response to chronic Aβ exposure.

## Graphical abstract

Alzheimer's disease (ad) is the most common cause of dementia, affecting over 35 million people worldwide. A key feature of ad is the loss of synapses—the connections between brain cells—which strongly correlates with cognitive decline. We sought to investigate the role of astrocytes in mouse organotypic cultures challenged with brain homogenate (BH) from ad patients to model early Aβ insult. Our results indicate that Aβ aggregates present in Aβ^+^ BH are responsive for increased synaptic activity, externalisation of signal lipid phosphatidyl serine (ptdSer) and synapse loss. We propose that glutamate toxicity due to hyperactivity can, at least in part, mediate the observed synapse loss. Astrocytes were found to prevent synapse loss and reduce ptdSer exposure in their proximity, but had no effect on activity reduction, suggesting that their effect lies downstream of changes in activity and involves glutamate removal via astrocytic glutamate transporters.

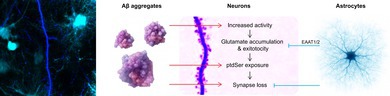

**Abbreviations**
AAV: adeno‐associated virus
ACET: glutamate transporter inhibitor
ACSF: artificial cerebrospinal fluid
ad
: Alzheimer's disease
APP: amyloid precursor protein
Aβ: amyloid beta
BFP: blue fluorescent protein
BH: brain homogenate
CA1: cornu ammonis area 1
CA3: cornu ammonis area 3
DG: dentate gyrus
DIV: days in vitro
EC: entorhinal cortex
ECFP: enhanced cyan fluorescent protein
EGFP: enhanced green fluorescent protein
ELISA: enzyme‐linked immunosorbent assay
GECI: genetically encoded calcium indicator
GFAP: glial fibrillary acidic protein
iACSF: imaging artificial cerebrospinal fluid
LMER: linear mixed‐effects regression/model
MCI: mild cognitive impairment
MOHSCs: mouse organotypic hippocampal slice cultures
ptdSer: phosphatidyl serine
REML: restricted maximum likelihood
ROI: region of interest
TBOA: TFB‐TBOA (glutamate transporter inhibitor)
WT: wild type

## 1 Introduction

Alzheimer's disease (ad) is a neurodegenerative condition that causes progressive cognitive decline with prominent early memory deficits (Jack et al. 2010). From a neuropathological perspective, ad is characterised by the progressive accumulation of aggregated amyloid beta (Aβ) in extracellular plaques, intracellular tau aggregates in neurofibrillary tangles, neuroinflammation in the form of accumulation of reactive astrocytes and microglia, and neurodegeneration (Thal et al. 2002).

Although advanced ad stages are characterised by extensive neuronal loss and brain atrophy, synapse loss can be observed decades before symptom onset and a clinical diagnosis of ad (Tzioras, McGeachan, et al. 2023). Reduction in synapse number seems to be prodromal of clinical diagnosis and has been reported in people with mild cognitive impairment (MCI) or early ad (Scheff et al. 2006). In people with both MCI and ad (DeKosky and Scheff 1990; Fang et al. 2024), synapse density strongly correlates with cognitive performance, particularly in mnemonic tests involving the activation of the medial temporal lobe (Terry et al. 1991; Scheff et al. 2006). In humans and mouse models that develop amyloid plaques due to expression of familial ad causing gene variants, synapse loss is particularly pronounced around plaques (Spires et al. 2005; Spires‐Jones et al. 2007; Koffie et al. 2009; Tzioras, McGeachan, et al. 2023; Colom‐Cadena et al. 2024; Koffie et al. 2012; Jackson et al. 2019; Colom‐Cadena et al. 2023), with soluble forms of Aβ being the most toxic in inducing synapse loss (Koffie et al. 2012; Spires‐Jones and Hyman 2014; Pickett et al. 2016; Busche and Hyman 2020).

Changes in brain activity are believed to occur around the time of onset of synapse degeneration. Several studies have reported an increase in brain activity in MCI subjects when performing hippocampal dependent mental tasks, whereas ad patients typically display a significant decrease in activity compared with healthy controls (Dickerson et al. 2005; Celone et al. 2006; Yassa et al. 2010; Putcha et al. 2011). Hyperconnectivity was reported in amyloid‐positive individuals with low tau burden, whereas hypoconnectivity emerged as tau burden increased, supporting the idea that this shift occurs in preclinical stages (Schultz et al. 2017). Consistent with this interpretation, elevated amyloid burden in older people without dementia has been linked to abnormal hyperactivity and elevated functional connectivity (Sperling et al. 2009; Cui et al. 2025). Soluble Aβ derived from ad patients' brain tissue impaired LTP induction but enhanced LTD in hippocampal slices and memory recall in rats receiving intraventricular injections (Shankar et al. 2008). Different mouse lines expressing human APP (hAPP) bearing various familial mutations, alone or in conjunction with PS1_dE9_, display neuron hyperexcitability and propensity to seizures (Palop et al. 2007; Minkeviciene et al. 2009). In similar ad mouse models, clusters of hyperactive neurons were found around plaques and display calcium dysregulation (Busche et al. 2008; Kuchibhotla et al. 2008). Aberrant calcium activity has also been reported at the level of individual synapses and dendrites (Bai et al. 2017).

Although cell‐autonomous effects likely play a role in synapse loss, glial cells have been progressively recognised as key mediators of neural health and toxicity in ad (Henstridge, Hyman, and Spires‐Jones 2019; Henstridge, Tzioras, and Paolicelli 2019; Tzioras, McGeachan, et al. 2023). Genetic studies have identified changes in a number of genes expressed in microglia and astrocytes as strong ad risk factors (Grubman et al. 2019; Bellenguez et al. 2022; Yang et al. 2023). A large number of ad risk gene variants are expressed in microglia, and the role of microglia in synaptic pruning, which is well known during brain development, has been suggested to act in a complement‐dependent manner in Alzheimer's models (Bartels et al. 2020). For these reasons, the field of synapse–glia interactions in ad has largely focused on microglia, whereas astrocytes have been relatively understudied. However, the strongest genetic modifier of sporadic ad risk, *APOE*, is highly expressed in astrocytes (Blumenfeld et al. 2024), and astrocytes can directly modulate synaptic function (Noriega‐Prieto and Araque 2021), arguing for the importance of a better understanding of astrocyte‐synapse interactions in the pathology.

Although much work has focused on the role of astrocytes in clearing pathological proteins from the brain, these cells are also crucial for synaptic function, with processes interacting directly with synapses providing metabolic support. A few studies of astrocyte‐synapse interactions have been conducted in cultured cells. These studies have produced conflicting results on astrocyte modulation of excitatory neurotransmission, some suggesting a protective role and some showing exacerbation of synaptic dysfunction by astrocytes (Talantova et al. 2013; Song et al. 2022).

Our previous work, employing post‐mortem human brain samples and cultured cells challenged with human post‐mortem ad synaptoneurosomes, demonstrated that astrocytes can remove synapses by phagocytosis, particularly in advanced ad cases (Tzioras, Daniels, et al. 2023). This complemented other studies showing that astrocytic profiles change with ad progression or amyloid pathology in mice, and reactive astrocytes can display mixed neuroprotective and neurotoxic features in response to Aβ and tau (Liddelow et al. 2017; Jiwaji et al. 2022; Serrano‐Pozo et al. 2024); for instance, astrocytes can actively clear Aβ deposits (Wyss‐Coray et al. 2003). Astrocytes also contribute to homeostatic synapse pruning in physiological conditions and have the ability to sense and modulate synaptic activity; for example, through the exposure of glutamate receptors at tripartite synapse processes (Noriega‐Prieto and Araque 2021).

It remains unknown whether Aβ‐induced hyperactivity in individual synapses leads to the loss of the synapse, and whether astrocyte interactions with individual synapses protect them from hyperactivity or degeneration downstream of Aβ. We address these questions in organotypic mouse hippocampal slices challenged with soluble Aβ contained in brain homogenate (BH) derived from ad patients' brains. Using soluble human ad BHs to challenge living brain tissue is a powerful tool to examine the pathological effects of soluble Aβ in a disease relevant brain context. Crucially, proteins derived from brain tissue have been secreted and oligomerized in the diseased brain in presence of co‐factors relevant to the pathology such as apolipoprotein E. We have previously observed that Aβ‐containing homogenate caused a reduction in the expression of synaptic genes when applied to human induced pluripotent stem cell‐derived neurons, and a loss of presynaptic puncta in living human brain slice cultures (King et al. 2023; McGeachan et al. 2025). Here, we combine this human‐relevant challenge with structural and functional imaging of synapses in organotypic slice cultures from mice with astrocytes labelled with a cyan fluorescent protein to test the hypothesis that astrocyte interactions modulate the synaptic response to Aβ.

## 2 Materials and Methods

### 2.1 Animals

C57BL/6J (wild‐type, WT) or GCFD mice were housed in groups in a 12/12 reverse light/dark cycle with food and water ad libitum. Pups of both sexes were humanely culled on postnatal days 5–8 to prepare organotypic slice cultures. The transgenic GCFD line was described in (Bai et al. 2013) and expresses the cyan fluorescent protein ECFP under the human *Gfap* promoter and is maintained on the FVB/N background. Both heterozygous ECFP^+/−^ and homozygous ECFP^+/+^ were used. Genotyping of pups was performed on tail tip samples by Transnetix with eGFP primers. Animal experiments were conducted in compliance with national and institutional guidelines including the Animals [Scientific Procedures Act] 1986 (UK), the Council Directive 2010/63EU of the European Parliament and the Council of 22 September 2010 on the protection of animals used for scientific purposes and had full Home Office ethical approval.

### 2.2 Mouse Organotypic Hippocampal Slice Cultures (MOHSCs)

MOHSCs were prepared as described in (Durrant 2020). Briefly, P5‐8 mouse pups were sacrificed by cervical dislocation. Brains were removed and transferred into 95%O_2_/5%CO_2_ ice‐cold, sterile filtered dissection medium: 87 mM NaCl (Sigma S7653), 2.5 mM KCl (Sigma P9333), 25 mM NaHCO_3_ (Sigma S5761), 1.25 mM NaH_2_PO_4_ (Sigma S5136), 25 mM glucose (Sigma G7021), 75 mM sucrose (Sigma S0389), 7 mM MgCl_2_ (Sigma M2393), 0.5 mM CaCl_2_ (Sigma C7902), 1 mM sodium pyruvate (Sigma P5280), 1 mM sodium ascorbate (Sigma A40034), 1 mM kynurenic acid (Sigma K3375), 1% Pen/Strep (50 U/mL, Thermo Fisher 15070063). Horizontal slices 350‐μm in thickness were cut with a Leica VT1000S vibratome in 95%O_2_/5%CO_2_ ice‐cold dissection medium. Hippocampal areas including a portion of the entorhinal cortex were microdissected with sterile needles and transferred to a falcon tube with fresh dissection medium. Slices were plated on 30 mm PTFE 0.4 μm pore sizes membrane inserts (Millicell PICM0RG50, Sigma‐Aldrich) in P35 tissue culture dishes (Greiner) with 1 mL MOHSC culture medium without phenol red: 50% v/v MEM (Thermo 51200038), 0.5% v/v Glutamax (Thermo 35050061), 12.5 mM HEPES (Gibco 15630080), 25% v/v heat‐inactivated Horse Serum (Thermo 26050070, lot 2554563), 18% EBSS (Thermo 14155063), 1.8 mM CaCl_2_ in EBSS (Sigma C7902), 0.324 mM MgCl_2_ in EBSS (Sigma M2393), 0.146 mM D‐glucose in EBSS (Sigma G7021), 0.5 mM ascorbic acid (Sigma A4544), 50 U/mL Pen/Strep (Thermo Fisher 15070063), 6 U/mL nystatin (Sigma N1638). For imaging experiments, slices were grown on square inserts cut from Biopore Membrane, hydrophilic PTFE, pore size 0.4 μm (Merk Millipore BGC00010, approximately 5–7 mm per side) on the surface of Millicell inserts. MOHSC were grown at 37°C, 5% CO_2_, 100% humidity. Medium was changed initially within 24 h of initial plating, after 4 days and once a week thereafter. From Day 1 onwards, 0.2 μM ACET (Tocris 2728) was included in the culture medium to reduce epileptogenic activity (Atanasova et al. 2023). Slices used in experiments were used between 2 and 5 weeks since plating.

### 2.3 Transgene Delivery

On day in vitro (DIV) 1 or 2, slices were infected in culture medium with adeno‐associated viruses (AAVs) diluted to desired concentration. A 3 μL drop of the virus solution was delivered on top of each slice, then they were returned to the incubator. Cre‐dependent transgenes were delivered via AAVs in conjunction with low‐titre Cre AAVs in concentrations determined in pilot experiments. The following AAVs were used in this study: pAAV.hsyn. Cre‐P2A‐Tdtomato (AAV9) (Addgene #107738‐AAV9, 2.6 10^13^ GC/ml), pENN.AAV.hsyn. Cre.hGH (AAV9) (Addgene #105555‐AAV9, 2.2 10^13^ GC/ml), pAAV.syn.flex.jGcamp7b.WPRE (AAV1) (Addgene #104493‐AAV1, 1.4 10^13^ GC/ml), pAAV.syn.flex.gcamp6s.WPRE.SV40 (AAV9) (Addgene #100845‐AAV9, 2.4 10^13^ GC/ml), pAAV.syn.flex.jGcamp8s.WPRE (AAV9) (Addgene #162377‐AAV9, 2.7 10^13^ GC/ml), pAAV‐Ef1a‐DIO‐GFP (AAV1) (Addgene #50457‐AAV1, 2.2 10^13^ GC/ml), pAAV‐Ef1a‐DIO‐mScarlet (AAV1) (Addgene #131002‐AAV1, 2.1 10^13^ GC/ml) and pAAV‐Ef1a‐Con/Foff 2.0‐BFP (AAV9) (Addgene #137130‐AAV8, 2.1 10^13^ GC/ml). Cre AAV (#104493) was diluted 1:1000 in the bleaching experiment and 1:10,000 elsewhere, whereas other AAVs were diluted 1:5 to 1:10 depending on the experiment. In PsVue experiments, Cre AAV (#105555) was diluted 1:4000 and BFP AAV (#131002) 1:10.

### 2.4 Live Imaging

Imaging was performed on a Leica DM6 microscope equipped with a TCS/SP8 system and a tunable multiphoton laser Chameleon (Coherent). Imaging was performed in a heated box at 37°C. Slices were maintained in constant imaging artificial cerebrospinal fluid (iACSF) heated to 37°C and constantly oxygenated with 95%O_2_/5%CO_2_. iACSF composition is 126.5 mM NaCl (Sigma S7653), 3.5 mM KCl (Sigma P9333), 2.5 mM CaCl_2_ (Sigma C7902), 1.3 mM MgCl_2_ (Sigma M2393), 1.2 mM NaH_2_PO_4_ (Sigma S5136), 26 mM NaHCO_3_ (Sigma S5761), 10 mM glucose (Sigma G7021), 0.4 mM Trolox (Sigma 64847) (Tashiro et al. 2006; Ishikawa et al. 2020). Biopore membrane inserts with the slices were carefully transferred to the imaging chamber (Warner Instruments RC‐27LD, Biochrom cat 641532/641551, sealed with silicone onto a glass coverglass—Biochrom 64‐0710) in a sterile tissue culture hood, primed with warm iACSF. The slice was maintained in place with the use of an imaging anchor (Warner Instruments 640258), taking care not to damage the slice with the threads. We typically removed one of the threads with a blade so that the remained threads only contacted the surface of the Biopore membrane but not the slice directly. The imaging chamber was positioned under the microscope objective onto a motorised stage (Scientifica) with an appropriated adaptor (Warner Instruments PM‐7D, Biochrom cat 641530/642411). Slices were kept submerged under constant oxygenated iACSF perfusion (approximate flow rate 1 mL/min) via a custom‐built system connecting polystyrene tubing (Warner Instruments PE‐160, Biochrom cat 640756) to STA‐PURE PCS pump tubing (Watson Marlow 961.0048.016) with silicone tubing. Forced flow (both inflow and outflow) was regulated with 120S/DV peristaltic pumps (Watson Marlow 010.6131.DA0). Outflow tubing was connected to an L‐shaped suction tube (Warner Instruments 641406). Images were acquired with a 2.5× objective (HC PL FLUOTAR 2.5×/0.07, Leica Microsystems) and a 25× immersion objective (HC FLUOTAR L 25×/0.95 W, Leica Microsystems, cat. 506374).

### 2.5 Structural and Calcium Imaging

Imaging areas were selected using a 2.5× objective and the relative position in the slice recorded with an epifluorescence camera (DFC7000T). Selected neurons were imaged with a 25× objective with 700 Hz bidirectional scan speed at 1024 × 1024 pixels; 1 μm step z‐stacks were taken. Second‐to‐third order dendrites in the CA1 stratum radiatum region, located in the imaging plane, were randomly selected and their position annotated for later retrieval. 1024 × 1024 pixel images of selected fields with a 10× digital zoom were taken to record the GCaMP, mScarlet and ECFP fluorescence (pixel size of 43.29 nm). The imaging field was rotated to position the selected dendrite along the x axis and centred on the y axis of the image. The laser was sequentially tuned to 800, 850 and 950 nm wavelengths to image the three fluorophores. Image dimension was then reduced to 144 × 50 pixels (pixel size 309.69 nm, frame speed 20.69 Hz) and 60‐s recordings of GCaMP fluorescence were performed at 20 Hz (50 ms interframe interval). Two to three recordings were performed, spaced by at least 1 min. Imaging was performed on a Thorlabs air table connected to compressed air to mechanically isolate the microscope from the environment.

#### 2.5.1 Longitudinal Analysis of Spine Loss

For structural imaging, only EGFP or mScarlet channels were recorded at high resolution. Slices at 14–21 DIV were used in this experiment. Slices were transferred to the imaging chamber and let equilibrate under the microscope for 5–10 min. Selected dendrites were imaged as described above (baseline) with the following modifications: 5‐μm stacks were taken (z‐step: 0.5 μm) with 7.5× digital zoom. Slices were then returned to the tissue culture hood and transferred back onto the original petri dish, which was maintained in the incubator during imaging. Treatment was started (see below) by replacing 25% of the culture medium with either BH (mock‐ or Aβ‐immunodepleted) or ACSF, and by adding 3 μL of the same solution on top of the slice. After 24 h (Day 2), the imaging session was repeated by imaging the same dendrite with the same setup as Day 1. Care was taken to match the field of view as closely as possible.

#### 2.5.2 Longitudinal Analysis of Synapse Activity and Spine Loss

For calcium imaging experiments, both structural, high‐resolution images (GCaMP, mScarlet and ECFP channels) and high‐speed recordings of GCaMP fluorescence were performed. First, we recorded the baseline activity and dendrite structure before treatment (baseline, or Day 1 PRE); then we started the challenge of the slices with either BH (mock‐ or Aβ‐immunodepleted), as described above. Slices were incubated for 2 h in the incubator, then imaged again (2 h POST). Slices were then returned in the incubator and imaged a third time at 24 h (24 h POST).

#### 2.5.3 Comparison of GCaMP Variants

GCaMP6s, jGCaMP7b and jGCaMP8s were initially compared in parallel. Live imaging was performed as above with the following parameters: digital zoom 10×, image size 512 × 110 pixels, 1 min recordings at 10 Hz; scan speed 700 Hz. Slices used for these experiments were grown as described above but in the absence of ACET.

#### 2.5.4 Longitudinal Analysis of ptdSer Exposure

PsVue550 (Molecular Targeting Technologies P‐1005) was dissolved in ACSF to 1 mM. Before each imaging session, PsVue550 was diluted in pre‐warmed imaging ACSF to 10 μM, and 5 μL was delivered on top of MOHSC and incubated at 37°C for 5 min. MOHSC were then washed in imaging ACSF and transferred to imaging chamber as described above. BFP was excited at 800 nm, whereas ECFP and PsVue550 were excited at 850 nm. Selected dendrites were imaged as described above: 5‐μm stacks were taken (z‐step: 0.5 μm) at 2048 × 2048 pixel with digital zoom 5×. Treatment with BH was identical to what done in the calcium imaging experiment, with the only exception that baseline (PRE), 2 h (POST) and 24 h imaging were all preceded by PsVue staining.

#### 2.5.5 Longitudinal Analysis of Synapse Activity and Spine Loss With Glutamate Transporters Inhibitors

The experiment was conducted as described above, with 10 nM TFB‐TBOA (in text, TBOA; Tocris 2532) or vehicle as a control added to the slice medium after baseline (PRE) recording. This yielded four treatment groups: Aβ^+^ TBOA^−^, Aβ^+^ TBOA^+^, Aβ^−^ TBOA^−^ and Aβ^−^ TBOA^+^. TBOA^+^ slices were imaged as above in iACSF with 10 nM TBOA.

### 2.6 BH Preparation

BH from temporal cortex of people who died with AD was prepared as described in Colom‐Cadena et al. (2024) with modifications. Two BH preparations were used in this work. The first preparation was used in all experiments except those detailed in Figure 5 and related supplementary figures. Data presented in Figure 5 and related supplementary figures (PsVue experiment) was collected using both preparations. The batch used was included as factor in the statistical analysis and was found to not have a statistical impact. Details of human cases used in the two preparations are reported in Tables 1 and 2, respectively. Human brain tissue was weighed and homogenised with a Dounce homogeniser in ice‐cold artificial cerebrospinal fluid (ACSF) (2 g in 10 mL) and placed in a low protein binding 15 mL tube (Thermo Fisher, 30122216). ACSF composition was 124 mM NaCl (Sigma S7653), 2.8 mM KCl (Sigma P9333), 1.25 mM NaH_2_PO_4_ (Sigma S5136), 26 mM NaHCO_3_ (Sigma S5761), pH 7.4, supplemented with EDTA‐free protease inhibitor (1 tablet/10 mL Roche 11836170001). The tissue was solubilised on a roller for 30 min at 4°C, then centrifuged at 2000 RCF for 10 min to remove large, insoluble debris. The supernatant was transferred to ultracentrifuge tubes (Beckman 355647) and centrifuged at 200,000 RCF for 110 min. The supernatant fraction containing soluble Aβ forms, was then transferred to a Slide‐A‐Lyser G2, 2K MWCO 15 mL dialysis cassette (Thermo Fisher 87719) and dialysis against ACSF (without protease inhibitors) for 3 days at 4°C to remove salts from the homogenate. ACSF was exchanged every 24 h. After dialysis, the BH was divided into low protein binding 15 mL tubes for immunodepletion. Protein A agarose (PrA) beads (Thermo 20334) were washed three times in ACSF with protease inhibitors. To create Aβ− treatment samples, Aβ was immunodepleted by adding 20 μL 4G8 antibody (Biolegend, 800711) and 30 μL of reconstituted PrA bead per 1 mL of homogenate. To create Aβ^+^ samples, homogenate was “mock‐immunodepleted” with 20 μL control anti‐GFP antibodies (DSHB, DSHB‐GFP‐12A6) and 30 μL of reconstituted PrA bead per 1 mL of homogenate. Aβ‐ and mock‐immunodepletion was performed at 4°C for 3 days. On Days 2 and 3, PrA‐antibody complexes were pelleted at 4°C for 5 min at 2500 RCF before adding fresh volumes of antibody and PrA to the supernatant. On the fourth day, only 30 μL per 1 mL were added to the supernatant to remove excess antibody and incubated 4°C for 2 h, then centrifuged for 5 min at 2500 RCF. The cleared BH was aliquoted in low‐bind tubes and stored at −70°C. Fractions of Aβ‐ and mock‐immunodepleted BH were analysed for Aβ1‐40 and Aβ1‐42 content with sandwich ELISA kits KHB3481 (Aβ40), KHB3441 (Aβ42) (Thermo Fisher). In the first batch, in the mock‐immunodepleted BH, the Aβ40 concentration was 227.2 pg/mL (52.5 pM), and Aβ42 was 140.6 pg/mL (31.1 pM). In contrast, in the Aβ‐immunodepleted BH, the Aβ40 concentration was 76.1 pg/mL (17.6 pM), whereas Aβ42 was below the detection limit in the undiluted fraction. In the second batch, Aβ40 and Aβ42 concentrations were respectively 452.9 pg/mL (104.6 pM) and 54.8 pg/mL (12.14 pM) in the mock immunodepleted BH, and 75.6 pg/mL (17.5 pM) and 4.1 pg/mL (0.91 pM) in the Aβ‐immunodepleted BH. Total human Tau content was 1.94 μg/mL and 1.31 μg/mL in Aβ^+^ and Aβ^−^ ad BH, respectively (first batch). We quantified the content of the following phosphorylated tau forms: Human tau pT217 12.50 ng/mL, Human tau pT231 703.38 ng/mL, Human tau pS199 861.79 ng/mL from Aβ^+^ ad BH (second batch). The following ELISA kits were used to determine the content: Human Total Tau KHB0041 (Invitrogen), Human tau pT217 AB318936 (Abcam), Human tau pT231 AB322367 (Abcam) and Human tau pS199 AB323734 (Abcam).

**TABLE 1:** Details of human donors in the first BH batch.

Diagnosis	Case ID	Age	Sex	PMI (h)	Braak stage	Thal stage	ApoE	Area	Approximate weight (g)
Alzheimer's disease	BBN001.35535	83	F	95	VI	4	ε3/ε4	BA37, BA38	3.24
Alzheimer's disease	BBN001.37165	70	M	7	VI	5	N/A	BA37, BA38, BA20/21	4.38
Alzheimer's disease	BBN001.35851	73	M	46	VI	5	N/A	BA37, BA41/42	1.91
Alzheimer's disease	BBN001.36297	84	M	87	VI	5	ε3/ε4	BA41/42	2.24
Alzheimer's disease	BBN001.36105	72	M	56	VI	5	N/A	BA37, BA41/42	1.65
Alzheimer's disease	BBN001.33626	86	F	114	VI	5	N/A	BA37, BA38	2.02
Alzheimer's disease	BBN001.34156	53	F	70	VI	5	ε4/ε4	BA20/21	2.83
Alzheimer's disease	BBN001.30973	89	F	96	VI	5	ε3/ε4	BA37	2.17
Alzheimer's disease	BBN001.32929	85	F	80	VI	5	ε3/ε3	BA37	1.85
Alzheimer's disease	BBN001.37400	71	M	75	VI	5	ε3/ε4	BA38	1.29
Alzheimer's disease	BBN001.36689	92	M	75	VI	5	ε3/ε4	BA38, BA20/21	2.10

**TABLE 2:** Details of human donors in the second BH batch.

Diagnosis	Case ID	Age	Sex	PMI (h)	Braak stage	Thal stage	ApoE	Area	Approximate weight (g)
Alzheimer's disease	BBN001.37882	59	F	31	VI	5	ε3/ε3	BA20/21, BA38	2.71
Alzheimer's disease	BBN001.37808	76	M	34	VI	5	ε3/ε4	BA20/21	1.30
Alzheimer's disease	BBN001.37702	78	F	95	VI	5	ε3/ε4	BA20/21	1.25
Alzheimer's disease	BBN001.37667	94	F	65	VI	5	ε3/ε3	BA38	1.30
Alzheimer's disease	BBN001.37400	75	M	71	VI	5	ε3/ε4	BA20/21	1.07
Alzheimer's disease	BBN001.37165	70	M	7	VI	5	ε3/ε3	BA20/21, BA38	2.40
Alzheimer's disease	BBN001.36924	72	F	66	VI	5	ε3/ε3	BA20/21, BA38	3.76
Alzheimer's disease	BBN001.36840	69	F	57	VI	5	ε3/ε3	BA20/21, BA38	2.00
Alzheimer's disease	BBN001.35182	66	M	49	VI	5	ε3/ε3	BA38	2.11
Alzheimer's disease	BBN001.33698	90	F	76	VI	5	ε3/ε4	BA20/21	1.47
Alzheimer's disease	BBN001.29695	86	M	72	VI	5	ε3/ε4	BA20/21	1.40
Alzheimer's disease	BBN001.29135	90	M	73	VI	3	ε3/ε4	BA38	1.29
Alzheimer's disease	BBN001.26732	76	M	66	VI	3	ε3/ε4	BA20/21, BA38	2.26
Alzheimer's disease	BBN001.26500	81	M	83	VI	5	ε4/ε4	BA20/21	2.12
Alzheimer's disease	BBN_24668	96	F	61	VI	N/A	ε3/ε4	BA38	0.95
Alzheimer's disease	BBN_24526	79	M	65	VI	N/A	ε3/ε4	BA38	0.64
Alzheimer's disease	BBN_19690	57	M	58	VI	N/A	ε3/ε4	BA20/21, BA38	1.90
Alzheimer's disease	BBN_15259	87	F	28	VI	N/A	ε3/ε4	BA20/21, BA38	3.00
Alzheimer's disease	BBN_15258	65	M	80	VI	N/A	ε3/ε3	BA20/21, BA38	2.91
Alzheimer's disease	BBN_15810	73	F	96	VI	N/A	ε4/ε4	BA20/21	0.98

### 2.7 Human Tissue Donor Information

Brain samples were obtained from tissue donated from people fulfilling clinical and neuropathological criteria for AD. Clinical and neuropathological data were obtained from the Edinburgh Brain Bank. In some instances where consent was in place, genotyping of the *APOE* allele was conducted by the Edinburgh Clinical Research Facility (Genetics Core). Neuropathological stages were applied according to international recommendations (Braak et al. 2006; Montine et al. 2012). Details of the human cases are reported in Tables 1 and 2. Use of human tissue for post‐mortem studies has been reviewed and approved by the Edinburgh Brain Bank ethics committee and the ACCORD medical research ethics committee, AMREC (ACCORD is the Academic and Clinical Central Office for Research and Development, a joint office of the University of Edinburgh and NHS Lothian, approval number 10/S1103/10). The Edinburgh Brain Bank is a Medical Research Council funded facility with research ethics committee (REC) approval (HV‐016).

### 2.8 Histology

#### 2.8.1 Adult GCFD Tissue

Two GCFD^+/+^ (13 and 10 months old) males and two GCFD^+/−^ (2 months old) females were sacrificed with an overdose of pentobarbital and transcardially perfused with cold phosphate buffered saline (PBS) and 4% formaldehyde in PBS (Agar Scientific AGR1026). Brains were postfixed overnight and cryoprotected in 30% w/v sucrose in PBS. Sagittal sections of 40 μm thickness were cut with a cryostat and slices containing the dorsal hippocampus were stained. Briefly, slices were blocked in 10% normal donkey serum (NDS, Sigma D9663) 0.3% Triton X‐100 (Sigma X100) in PBS for 30 min. Slices were incubated overnight at 4°C with primary antibodies in 10% NDS 0.1% Triton X‐100 in PBS as indicated in text: chicken anti‐GFP 1:1000 (Abcam ab13970 lot GR261775‐3), rat anti‐GFAP 1:1000 (Thermo Fisher 130300 clone 2.2B10), rabbit anti‐S100β 1:1000 (Abcam ab41548), guinea pig anti‐NeuN 1:500 (SySy 266004). The following day slices were washed three times in PBS and incubated with secondary antibodies 1:200 in PBS for 2 h: anti‐chicken AlexaFluor488 (Thermo Fisher A11039 lot 1812246), anti‐rat AlexFluor647 (Thermo Fisher A21247 lot 2005938), anti‐rabbit AlexaFluor647 (Thermo Fisher A21244 lot 2086730), anti‐guinea pig Cy3 (Sigma AP108C). Slices were then incubated for 10 min in 2 μg/mL DAPI (Sigma D9542), washed three times in PBS and mounted in ImmuMount (Thermo Fisher 9990402).

#### 2.8.2 GCFD MOHSCs

MOHSCs were prepared as described above and maintained in vitro until DIV21, when they were washed in PBS before fixation with 4% PFA for 1 h. Following three PBS washes, slices were removed from their membrane inserts with a scalpel to produce free‐floating tissue samples. Slices were permeabilised with 0.3% Triton X‐100 in PBS for 20 min. The tissue was then placed in 1% citrate‐based antigen unmasking solution (Vector Laboratories H‐3300‐250) and pressure cooked for 2 min. After a single PBS wash, slices were placed in 5% horse serum (Gibco 26050070)/0.3% Triton X‐100 in PBS for 1 h. Primary antibody solution with chicken anti‐GFAP 1:1000 (Abcam AB4672) made in 5% horse serum/0.3% Triton X‐100 in PBS was added to the slices, which were incubated at 4°C for 48 h. Following 5× PBS washes, secondary antibody solution containing anti‐chicken AlexaFluor647 (Invitrogen A21449) made in 5% horse serum/0.3% Triton X in PBS was added to the slices, which were incubated at 4°C for 24 h. After a further 5× PBS washes and a single dH_2_O wash, slices were mounted in Prolong Glass mounting medium (Invitrogen P36984).

#### 2.8.3 Imaging

Slices were imaged with Leica TCS SP8 confocal microscope using oil objective HC PL APO CS2 63×/1.40NA with 1 AU pinhole. Laser lines 405, 488, 552 and 638 nm were used for DAPI and ECFP, AlexaFluor488, Cy3 and AlexaFluor647, respectively. ECFP endogenous fluorescence was integrated in the 450–550 nm range.

### 2.9 Data Analysis

#### 2.9.1 Analysis of Calcium Activity and Event Extraction

Recordings and images were processed in Fiji/ImageJ (Schindelin et al. 2012). First, image background was subtracted using a 50‐pixel radius rolling ball, and the resulting images were converted to 16‐bit. Recordings where a drift of the dendrite was observed, either in the x–y plane or the optical plane, were excluded from the analysis. Synapse identification was performed on projections of the recording, using the high‐resolution images as reference. Region of interests (ROIs) were drawn around visible spines and exported as ROI sets. Integrated intensity over time was exported as .csv with the ROI manager “Measure” tool. PRE recordings were analysed first. ROI sets for additional recordings (both PRE and POST treatment) were loaded onto the images and translated rigidly as needed (i.e., translated and rotated to account for differences in the exact alignment of the selected portion of the dendrite with the field of view during repeated imaging sessions). Minimal adjustments in the positions of the ROIs were performed using the projection of each recording as template. Extracted temporal profiles were analysed in Python. Briefly, baseline was estimated with a 10th order polynomial fit and subtracted. The resulting trace was denoised with Okada filter (four iterations) (Ishikawa et al. 2020) and converted into ΔF/F values. Events were automatically detected with CaImAn implementation of CNMFe/OASIS v.1.9.12 (Friedrich and Paninski 2016; Giovannucci et al. 2019) using function constrained_foopsi from caiman.source_extraction.cnmf.deconvolution on z‐scored normalised traces (sn **=** 10, method_deconvolution **=** 'oasis', optimize_g **=** 5, s_min **=** 2.5), effectively applying a threshold of 2.5σ for events to be considered. Event frequency was exported for each synapse ID (experiment, slice and ROI number) and merged with further information.

#### 2.9.2 Comparison of GCaMP Variants

Analysis was performed as above, with the following exceptions: Only one iteration of Okada filter was performed, and a threshold of *s* = 1.5 on z‐scored normalised traces was used for event detection. Events detected with CNMFe/OASIS were aligned and plotted as ΔF/F and z‐scored traces. Peak amplitude was calculated at peak time detected by CNMFe/OASIS on ΔF/F traces. The Bleaching profile was calculated by integrating the raw dendrite intensity in each frame for 1 min and normalising values on *t* = 0. When inspecting the bleaching profile, we often detected a sharp decline, likely due to an immobile fraction of the fluorophore, followed by a slow, steady decline. We therefore fitted the temporal profile with a biexponential decay using scipy.optimize.curve_fit. The Bleaching rate was defined as the difference (F_0_ − F_60_), where F_0_ is the average normalised intensity between Frames 5 and 10, and F_60_ is the average normalised intensity of the last five frames.

#### 2.9.3 Annotation of Synapse Survival and Proximity by Astrocytes

After background subtraction, maximum projections of stacks were performed and channels separated. Dendrites fields of view of required time points were rigidly aligned with Stackreg (Thévenaz et al. 1998), and spine survival was annotated using slice and synapse ID, comparing images from Days 1 and 2. Astrocyte proximity was determined by comparing high‐resolution the neuron channel (mScarlet) and the astrocyte channel (ECFP) from pre‐treatment imaging sessions. A positive astrocyte proximity status was considered if either there was a superposition of ECFP and mScarlet signals, or if the ECFP signal was detected within a 1‐synapse radius from the synapse ROI. Annotation was done blind to the condition.

#### 2.9.4 PsVue Signal Quantification

Channels were separated and processed independently as described above. Longitudinal registration was performed on the BFP channel equivalently to mScarlet channel as described above. If needed, registration of the 800 and 850 nm stacks was aligned with MultiStackReg using the common BFP channel, that is excited by 800 nm and, to a lesser extent, by 850 nm wavelength. PsVue550 signal was acquired in the red channel. First, image background was subtracted using a 50‐pixel rolling ball radius (Fiji/ImageJ); then, the sum projection of the stack was performed, and the background was subtracted again. ROIs were drawn on the BFP channel around dendritic spines, applied to the PsVue channel and the mean intensity was measured. In field measurements (Figure S7), the mean intensity in the whole image was quantified.

#### 2.9.5 Data Analysis

After annotation, event rate at various time points, astrocyte contact, synapse survival at 24 h were merged automatically with experimental information (treatment, animal sex) in Python. For each time point (Day 1 PRE, Day 1 POST), the average event rate for each synapse was calculated if multiple recordings were included. Absolute event rate (events per second) and relative changes were considered in the analysis. Relative change of activity after the first 2 h if treatment was calculated as ΔHz/Hz = (POST − PRE) / PRE. PsVue data was expressed similarly as relative change of activity after the first 2 h if treatment was calculated as ΔPsVue/PsVue = (POST − PRE) / PRE. Data were analysed with restricted maximum likelihood estimation (REML) linear mixed models (LMER) using animals and slices as random effect factors to avoid pseudoreplication using Python or R. To analyse the loss of synapses following 24 h of treatment, the model Percent_loss ~ Treatment*Sex + (1|animal, slice) + (1|fluorophore), with the fluorophore factor included as a smaller number of neurons expressing EGFP; the majority of the neurons in this analysis expressed mScarlet as filler. LMER models used are indicated in the text. For instance, for activity, activity change and survival, the LMER models were Activity ~ Aβ*Treatment_time*Sex + (1|animal, slice), Activity_change ~ Aβ*Survival + (1|animal, slice) and Activity_change ~ Aβ*Astrocyte*Sex + (1|animal, slice), and Fraction_surviving_synapses ~ Aβ*Astrocyte*Sex + (1|animal, slice), respectively. Post hoc differences were calculated in R with emmeans using the Tukey correction. Residuals and Q–Q plots were inspected, and normality was tested with the Shapiro–Wilk normality test.

#### 2.9.6 Software

R v4.4.3 with libraries lme4 v1.1‐37, lmerTest v3.1‐3, stargazer v5.2.3, emmeans v1.11.0, ggResidpanel v0.3.0 were used for data analysis. Python analysis was run in Jupyter notebook.

#### 2.9.7 Randomisation and Blinding

Brain slices were randomly assigned to treatment conditions, with slices from each mouse receiving all treatment conditions for each experiment as a within‐animal control. Event activity was analysed with minimal user intervention with an algorithm. In particular, event calculation was fully automated. Synapse detection, determination of synapse survival and synapse proximity to astrocytes were performed independently from each other using only the slice ID and ROI number as identification. The experimenter was blind to treatment and experimental conditions.

## 3 Results

### 3.1 ad Brain Derived Aβ Induces Spine Loss in Mouse Organotypic Slices

**FIGURE 1:**
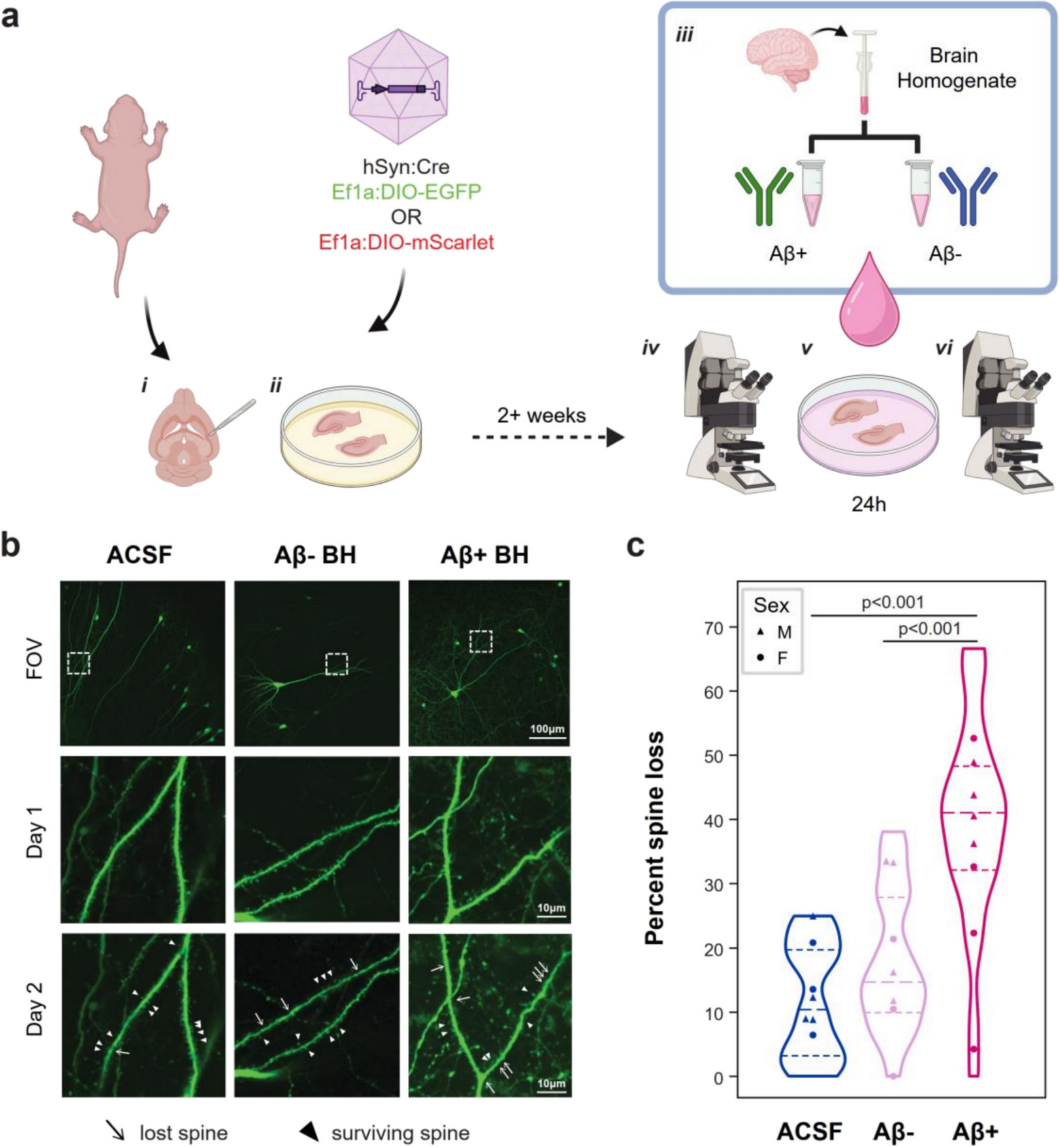
ad brain homogenate causes significant loss of synapses in organotypic mouse hippocampal slices*.* (a) Mouse organotypic hippocampal slice cultures (MOHSCs) were prepared (i) and infected with AAVs expressing soluble fluorescent proteins on day in vitro 1 (DIV1) (ii). After 2–4 weeks in culture, slices were first imaged (iv), then incubated with ACSF or brain homogenate from ad patients for 24 h (v). Brain homogenate was previously immunodepleted for Aβ (Aβ^−^ BH) or mock immunodepleted (Aβ^+^ BH) (iii). A second imaging session was performed on Day 2 (vi), and synapse loss evaluated comparing images at (iv) and (vi) time points. (b) Representative images showing the field of view (FOV) of whole neurons (top row), followed by higher‐magnification views of dendritic fields pre‐ (Day 1) and post‐treatment (Day 2). Dendritic areas correspond to the dashed box shown in the FOV images. Scale bars 100 μm for FOV and 10 μm for dendritic fields. (c) Quantification of spines lost after 24 h treatment as a percentage of spines detected on Day 1, and not on Day 2. Each point is an individual animal. Here and in all figures, males are represented by triangles and females by circles. <LMER % spine loss ~ Treatment*Sex + (1|animal, slice) + (1|filler); see text for details. Panel (a) created in BioRender, J. Tulloch (2026), https://BioRender.com/fy05caa.

To model early exposure to Aβ toxic species, we challenged MOHSCs with the soluble fraction of BH derived from ad patients (Aβ^+^ BH) (see Section 2.6 and Table 1). This fraction contains soluble Aβ forms such as Aβ1‐40 and Aβ1‐42 in the 100 and 10 pM range, respectively. First, we aimed to determine with live imaging if ad BH can cause synapse loss in MOHSC neurons. To this end, we expressed fluorescent markers in a sparse subset of pyramidal neurons in the MOHSC area corresponding to hippocampal CA1. We quantified longitudinal synapse loss by counting the number of dendritic spines disappearing between imaging sessions before and after 24 h of treatment. Second‐to‐third order dendrites were randomly selected from the apical dendritic arbour in the stratum radiatum. After the first imaging session (baseline), slices were incubated with either Aβ^+^ BH, Aβ^−^ BH (devoid of Aβ species by means of immunodepletion) or ACSF control. For consistency, Aβ^+^ BH was mock immunodepleted with neutral anti‐GFP antibodies (Figure 1a, see Section 2.6). Our results demonstrated that mock‐immunodepleted Aβ^+^ BH caused a significantly higher loss of synapses compared with control, Aβ^−^ BH and ACSF treatment. Spine loss under Aβ^−^ BH conditions was not significantly different from ACSF controls (*n* = 22 mice (10 F, 12 M), 61 dendrites (one neuron per slice), 4536 spines at baseline; linear mixed‐effects model (LMER) % spine loss ~ Treatment*Sex + (1|animal, slice) + (1|fluorophore); Treatment *F*(2,55) = 28.53, *p* < 0.0001; Sex *F*(1,7.37) = 1.43, *p* = 0.27. Tukey's post hoc comparison (Aβ^+^–ACSF) *t* = 6.19, *p* < 0.0001; (Aβ^+^–Aβ^−^) *t* = 4.98, *p* = 0.0001) (Figure 1b,c).

### 3.2 Multicolour Imaging of Synaptic Activity and Astrocyte–Neuron Interactions

We next asked whether the loss of synapses induced by Aβ^+^ BH was influenced by changes in synaptic activity or astrocyte proximity. To this aim, we performed calcium imaging experiments and quantified the frequency of synaptic events before and after Aβ^+^ BH challenge. Although calcium indicators to monitor somatic neural activity are well established (Cichon et al. 2020; Gobbo et al. 2022), synaptic events typically display low signal‐to‐noise levels even with the best performing genetically encoded calcium indicators (GECIs) (Yasuda et al. 2004). Furthermore, no systematic comparison has been performed in the literature. Synaptic transients have been imaged with individual indicators GCaMP6s (Chen et al. 2013; Cichon and Gan 2015), jGCaMP7 variants (in particular, 7b) (Dana et al. 2019) and jGCaMP8s (Zhang et al. 2023). We therefore aimed to compare these three variants in preliminary experiments by expressing Cre‐P2A‐Tomato and one of the three GECIs in a subset of CA1 pyramidal neurons (Figure S1a,b).

First, we evaluated the bleaching rate of the three variants when illuminated at two‐photon in WT MOHSCs (*n* = 32 dendrites from eight mice, Figure S1c). We observed similar bleaching rates between the three indicators (one‐way ANOVA *F*(2,29) = 1.62 *p* = 0.21, Figure S1d). We then compared synaptic transients detected during 60‐s recordings and considered individual synaptic events as the peaks in the deconvolved calcium trace (Giovannucci et al. 2019). We observed similar temporal profiles of the individual transients (Figure S1e). On average, jGCaMP7b showed higher ΔF/F peak amplitude compared with GCaMP6s and jGCaMP8s (LMER Peak ~ GECI + (1|animal); z(8 s) = −22.79 *p* < 0.001 z(6 s) = −14.86 *p* < 0.001, *n* = 4126 events from 12 dendrites/7 animals) (Figure S1f). We therefore selected jGCaMP7b as our calcium indicator of choice in our experiments (Figure S2).

**FIGURE 2:**
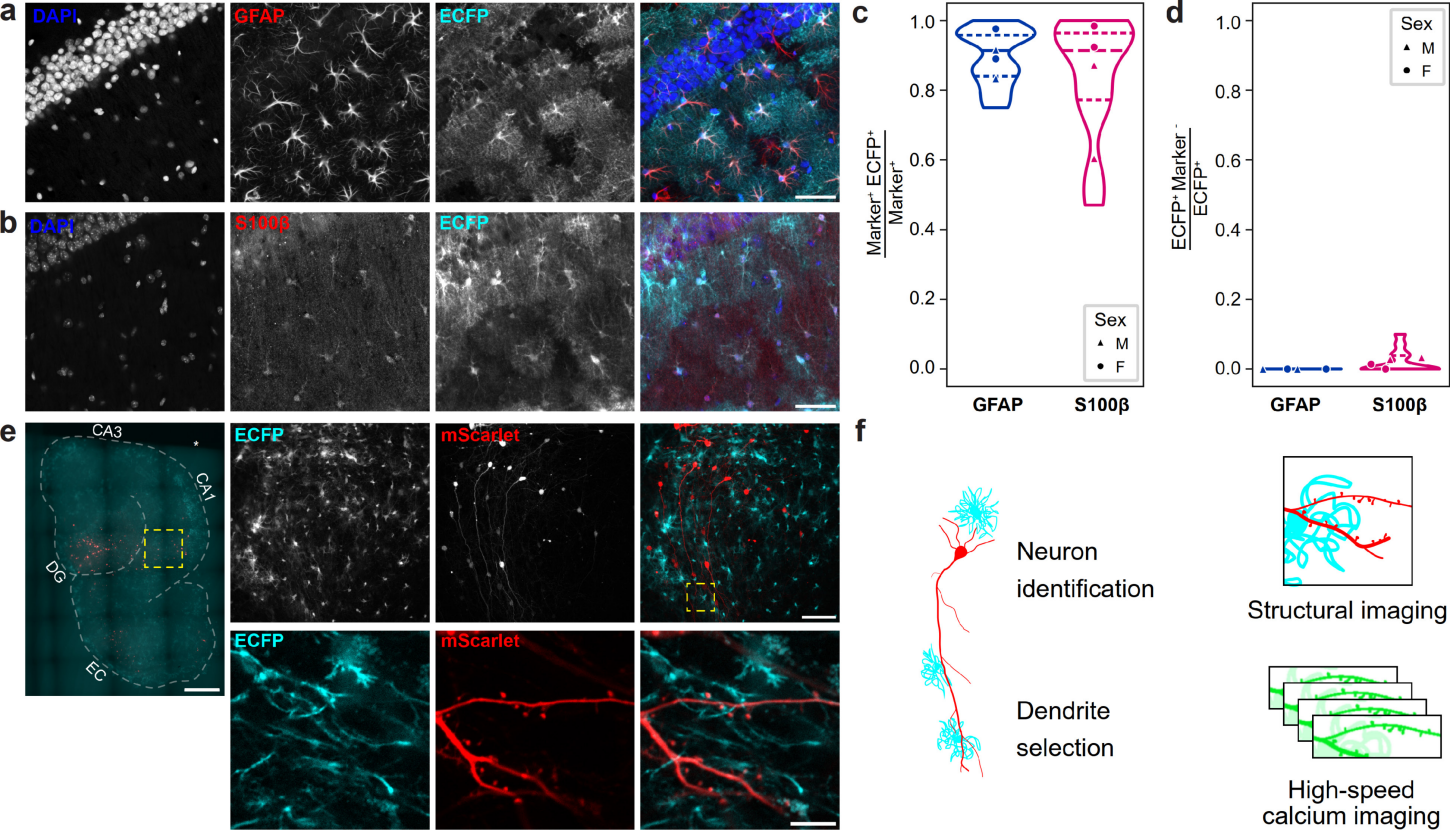
Three‐colour visualisation of astrocytes and neurons in culture. (a) Histological staining of hippocampal slices from GCFD animals showing the extensive overlap between ECFP (cyan in merge) and GFAP (red in merge) staining. Scale bar, 50 μm. (b) Histological staining as in (a) for ECFP (cyan) and S100β (red). (c) Quantification of ECFP overlap with GFAP and S100β markers expressed as fraction of GFAP or S100β cells. Points are individual animals. (d) Quantification of ECFP labelling of cells negative for GFAP and S100β markers expressed as fraction of ECFP cells. Points are individual animals. (e) Expression of ECFP (cyan) in astrocytes in live MOHSC and infected with AAVs expressing mScarlet (red) and jGCaMP7b. The profile of the slice is outlined for clarity, and the main hippocampal regions labelled. DG: dentate gyrus, CA3: cornu ammonis area 3, CA1: cornu ammonis area 1, EC: entorhinal cortex. Scale bar 500 μm. Panels on the right: top row, multiphoton images displaying ECFP (cyan in merge) and mScarlet channels (red in merge); corresponding field is marked in yellow on the slice image. Scale bar 100 μm. Bottom row, representative images for structural reconstruction of the dendrite and spine count displaying ECFP (cyan in merge) and mScarlet channels (red in merge). Scale bar 10 *μ*m. (f) Outline of imaging protocol.

To visualise astrocytes in living slices, we prepared MOHSC from the GCFD mouse line that expresses the enhanced cyan fluorescent protein (ECFP) controlled by the human *Gfap* promoter (Bai et al. 2013). The transgene is integrated in the genome and the line retains the two endogenous *Gfap* copies. We evaluated the percentage of ECFP^+^ astrocytes in this mouse line by staining histological hippocampal sections from adult GCFD animals (two males, two females, see Section 2.8) for endogenous astrocytic markers GFAP and S100β. We calculated that 90% of GFAP^+^ cells and 85% of S100β^+^ cells express ECFP (Figure 2a–c). Conversely, no other cell type was found to be ECFP‐positive (Figures 2d and S3). Similar to ex vivo analysis, the vast majority of astrocytes were found to be ECFP^+^ in MOHSCs derived from GCFD pups (Figure S4). We then co‐expressed jGCaMP7b (Dana et al. 2019) and mScarlet (Bindels et al. 2017) in a sparse subset of excitatory neurons by infecting slices with a combination of Cre‐dependent AAVs (Figure 2e, see Section 2). jGCaMP7b allowed us to record neuronal activity within individual dendritic spines, whereas the red fluorescent protein mScarlet was used as a marker to reconstruct the dendritic profile (Figure 2e,f). ECFP is a soluble protein filling the entire astrocyte, thus allowing a faithful reconstruction of astrocytes including the finest processes, unlike conventional GFAP staining and similar immunohistochemistry‐based methods (Figure 2a).

### 3.3 Aβ^+^ BH Treatment Induces an Increase in Synaptic Activity

**FIGURE 3:**
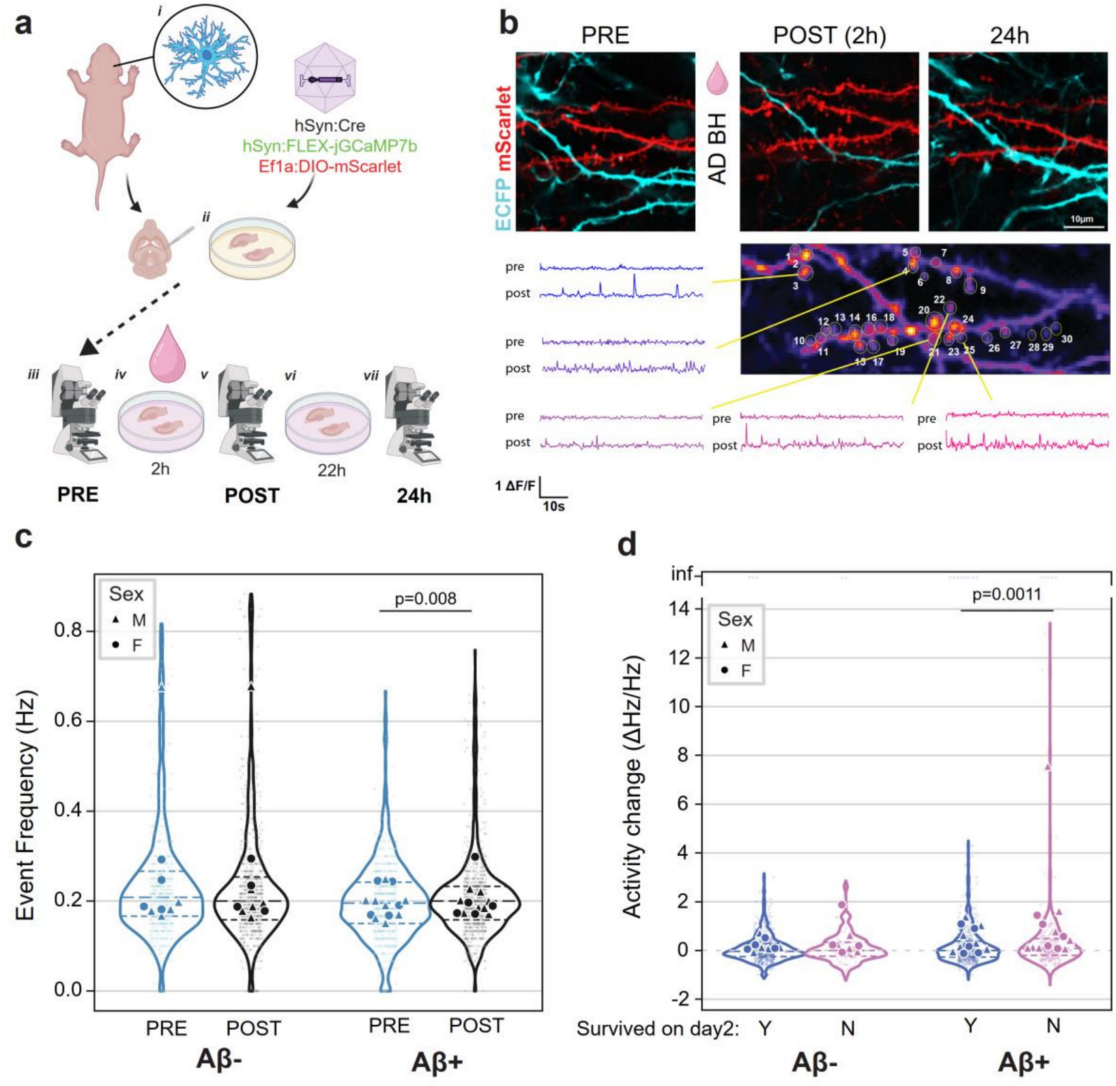
ad brain homogenate causes a significant increase in synaptic activity associated with synapse loss. (a) Outline of experiment. After extraction from GCFD pups (i), MOHSCs are infected with AAVs to express jGCaMP7b and mScarlet in a sparse neuron population (ii). Baseline activity and structural profile of selected dendrites are then imaged at baseline (iii), and at 2 h (v) after incubation with mock immunodepleted (Aβ^+^) or Aβ‐immunodepleted (Aβ^−^) brain homogenate (ad BH) (iv). Slices are then returned to treatment (vi) and imaged again at 24 h (vii). Created in Biorender, J. Tulloch (2026), https://BioRender.com/t7xfm11. (b) A typical example of longitudinal recording of the same dendrite before treatment (PRE), after 2 h incubation with ad BH (POST) and again the following day (24 h). Images are the overlap of the neuron/mScarlet (red) and astrocyte/ECFP (cyan) channels. Scale bar 10 μm. Below, projection of the GCaMP channel in false colours showing the regions identified as synapses (1–30). Displayed are representative ΔF/F traces of GCaMP fluorescence over 60 s before (pre) and after treatment (post). Scale bar 1 ΔF/F, 10 s. (c) Event frequency for detected synapses before (PRE) and after treatment (POST) with Aβ^+^ or Aβ^−^ BH. Small dots and violin plots are values from individual synapses, whereas large dots are animal averages. (d) Changes in event frequency after 2 h treatment with Aβ^+^ or Aβ^−^ BH, divided by synapses still present (Y) or lost (N) at 24 h. Large dots are animal averages, whereas small dots and violin plots are values from individual synapses. *Note:* A small number of infinite (inf) values occur where no events were detected in PRE recording and are displayed as “inf” on the y axis. LMER details are provided in text.

First, we measured the response of individual synapses to ad BH by comparing their baseline activity with the event frequency after 2 h of incubation (Figure 3a). We observed a significant increase of event frequency from baseline in synapses from slices incubated with Aβ^+^ BH, but not Aβ^−^ BH (Figure 3a,b, LMER Event rate ~ Treatment time (PRE/POST treatment)*Aβ*Sex + (1|animal, slice), Aβ *F*(1,40.96) = 3.05 *p* = 0.088 Aβ*Treatment Time *F*(1,2976.2) = 7.52 *p* = 0.006, Sex *F*(1,13.12) = 0.18, *p* = 0.68. Tukey post hoc comparison: (Aβ^−^| PRE–POST) z ratio = 0.95 *p* = 0.34, (Aβ^+^| PRE–POST) z‐ratio z = −3.044 *p* = 0.0023, *n* = 1513 spines from 18 animals). We confirmed the increased event rate after Aβ^+^ challenge in MOHSCs prepared from WT animals to rule out any effects of overexpressing ECFP in astrocytes on Aβ‐induced hyperactivity (Figure S5a,b, LMER Event rate ~ Treatment time (PRE/POST treatment) + (1|animal, slice), Treatment Time *z* = −4.89 *p* < 0.001, *n* = 136 spines from 5 slices/4 animals). There was no effect of astrocyte proximity on the change in synaptic activity, the main effect being determined by the treatment (Figure S6, LMER ΔHz/Hz ~ Aβ*Astrocyte + (1|animal, slice), Astrocyte *F*(1,1194.5) = 0.72 *p* = 0.39, Aβ *F*(1,41.59) = 4.28 *p* = 0.0448 Aβ*Astrocyte *F*(1,1194.5) = 2.13 *p* = 0.14). We also evaluated the synaptic activity at 24 h in a similar way in a subset of surviving synapses, showing a maintained increase in event rate in the Aβ^+^ BH, but not Aβ^−^ BH group (Figure S7a LMER Event rate ~ Treatment time (PRE/POST/24 h treatment)*Aβ + Sex + (1|animal, slice), Time *F*(2,1981.77) = 23.56 *p* < 0.0001 Aβ*Treatment Time *F*(1,1981.77) = 18.52 *p* < 0.0001, Sex *F*(1,20.23) = 1.61, *p* = 0.22. Tukey post hoc comparison: (Aβ^−^| PRE–POST) z ratio = 1.35 *p* = 0.21, (Aβ^−^| PRE–24 h) z ratio = −0.65 *p* = 0.79, (Aβ^+^| PRE–POST) z‐ratio *z* = −4.92 *p* < 0.0001, (Aβ^+^|PRE–24 h) z‐ratio *z* = −9.08 *p* < 0.0001 *n* = 675 spines from 18 animals). The change in activity at 24 h was higher for Aβ^+^ BH (Figure S7b, LMER ΔHz/Hz ~ Aβ*Sex + (1|animal, slice), Sex *F*(1,33.75) = 0.14 *p* = 0.70, Aβ *F*(1,33.75) = 3.09 *p* = 0.088 Aβ*Sex *F*(1,33.75) = 0.45 *p* = 0.51); again the effect of astrocyte proximity had no significant effect on the rate of change (Figure S7c, LMER ΔHz/Hz ~ Aβ*Astrocyte + Sex + (1|animal, slice) Sex *F*(1,35.95) = 0.27 *p* = 0.60, Aβ *F*(1,35.37) = 2.97 *p* = 0.09 Astrocyte *F*(1,644.35) = 0.20 *p* = 0.65). We therefore conclude that challenging MOHSCs with ad BH can cause a significant, Aβ‐dependent, activity increase in a subset of synapses.

### 3.4 Increased Activity in Spines Lost After Aβ^+^ BH Treatment

We then asked if there was any relationship between changes in activity measured at 2 h and the fate of the synapse at 24 h. We found that spines lost after 24 h of treatment with Aβ^+^ BH had on average a significantly higher change in activity measured after 2 h of treatment. The same was not observed for Aβ^−^ BH (Figure 3d; LMER ΔHz/Hz (Tukey transformed) ~ Aβ*Survival + (1|animal, slice), Survival F(1,1352.2) = 5.11 *p* = 0.024, Aβ *F*(1,59.32) = 3.17 *p* = 0.08 Aβ*Survival *F*(1,1352.2) = 1.46 *p* = 0.23, Tukey post hoc comparison: (Aβ^−^|Survived—Eliminated) z ratio = −0.062 *p* = 0.535, (Aβ^+^|Survived—Eliminated) z‐ratio z = −3.27 *p* = 0.0011, *n* = 1357 spines from 18 animals).

### 3.5 Astrocyte Proximity Is Associated With Spine Survival

**FIGURE 4:**
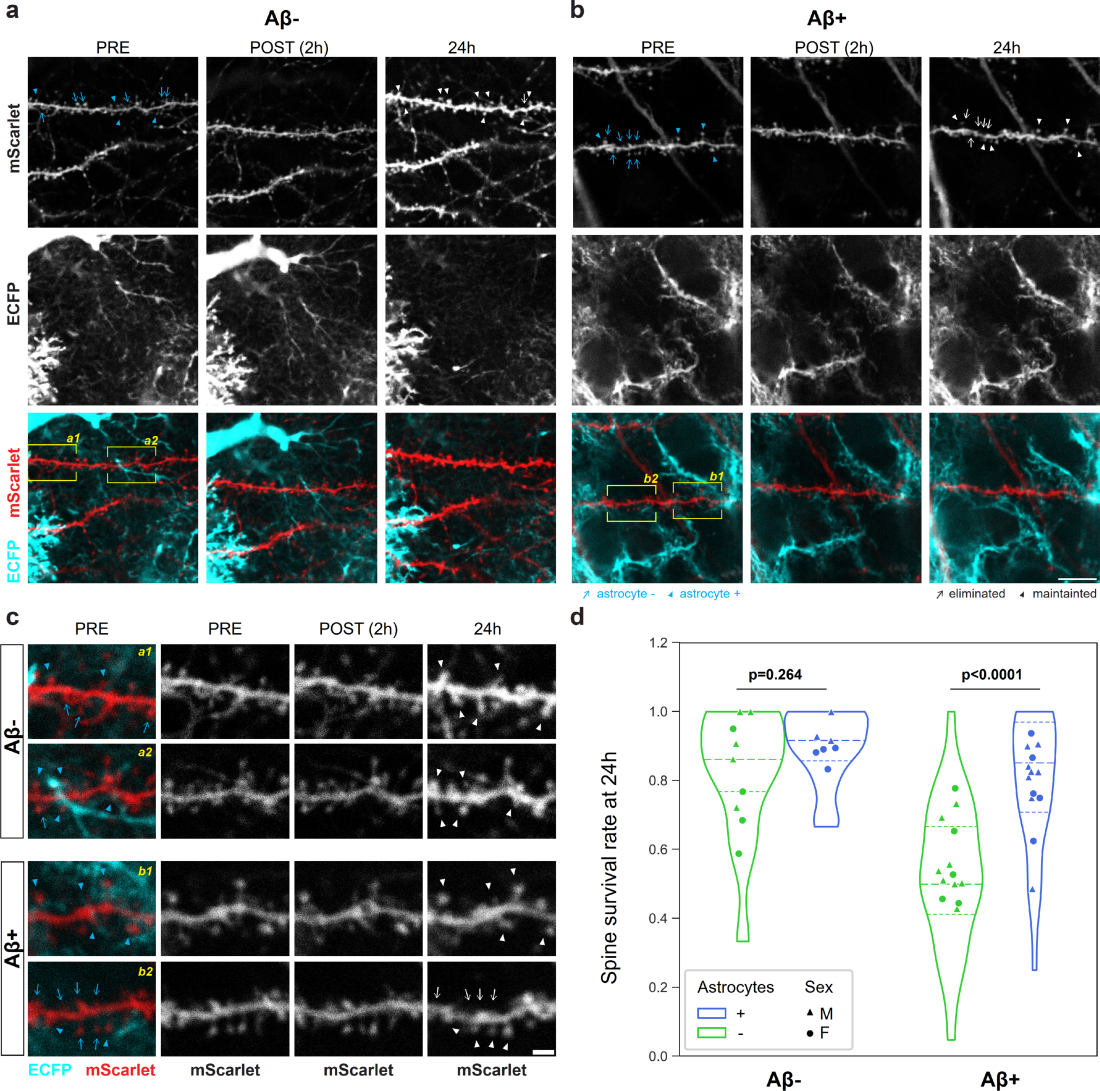
Relationship between synapse loss and astrocytes. (a, b) Representative longitudinal imaging of astrocyte/ECFP (cyan) and neuron/mScarlet (red) interaction after Aβ^+^ (a) or Aβ^−^ BH (b) treatment. Highlighted are synapses with or without astrocytes in their proximity (filled and empty cyan arrowhead, respectively), and maintained or lost ones (filled and empty white arrowhead, respectively). Scale bar, 20 μm. (c) Magnification of above images after registration. Corresponding areas in (a, b) panels are indicated in yellow. Scale bar, 2 μm. (d) Fraction of surviving spines after 24 h Aβ^+^ or Aβ^−^ BH treatment divided by astrocyte contact. Violin plots are individual dendrites, and dots are animal averages. LMER details in text.

We found that a significant factor in determining synapse survival was their association with astrocyte processes (Figure 4). We calculated the survival rate for each dendrite for synapses associated with astrocytes or without an astrocyte in their proximity (astrocyte positivity was defined as an astrocyte process that was within the radius of the spine head to the mScarlet‐positive dendritic spine head). Synapses with proximal astrocytic processes were significantly more likely to be maintained at 24 h in MOSHCs treated with Aβ^+^ BH (LMER Survival ~ Aβ*Astrocyte + (1|animal, slice) Aβ *F*(1,41) = 17.51 *p* = 0.00015 Astrocyte *F*(1,41) = 19.10 *p* < 0.0001; Aβ *Astrocyte *F*(1,41) = 6.37 *p* = 0.016, Tukey post hoc comparison: (Aβ^−^| Astrocyte yes—no) *z* = 1.14 *p* = 0.264 (Aβ^+^|Astrocyte yes—no) *z* = 5.94 *p* < 0.0001, *n* = 46 dendrites from 18 animals). Although motility of astrocytic processes was observed in the fields around the dendrites (Figures 4 and S8), we did not observe a significant loss or discrepancy in the astrocyte number after 24 h incubation with Aβ^+^ or Aβ^−^ BH (Figure S9, LMER N ~ Aβ + Sex + (1|animal) Aβ *F*(1,7) = 0.19 *p* = 0.86).

### 3.6 Astrocyte Proximity Is Associated With Reduced Externalisation of Phosphatidyl Serine

**FIGURE 5:**
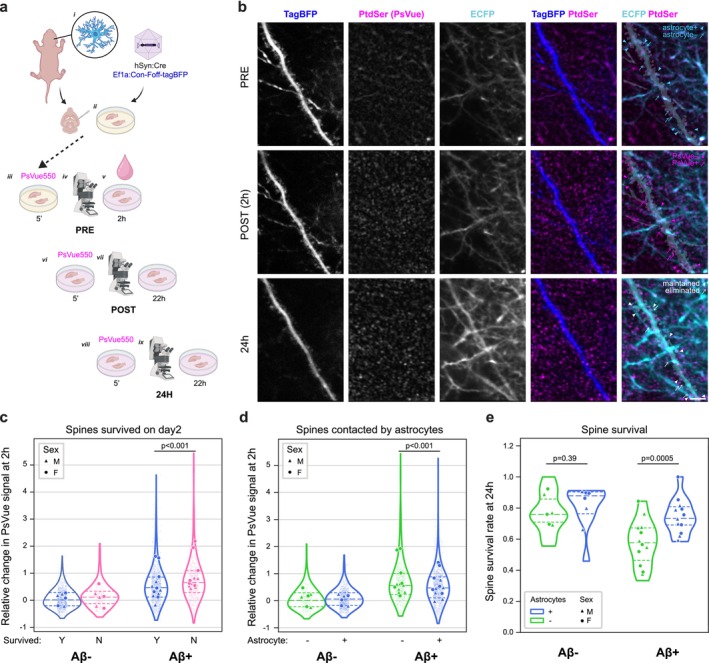
Relationship between synapse loss and phosphatidyl serine exposure. (a) Outline of experiment. After extraction from GCFD pups (i), MOHSCs are infected with AAVs to express tagBFP in a sparse neuron population (ii). Phosphatidyl serine (ptdSer) is visualised with PsVue550 staining (iii, vi and viii) before imaging sessions of selected dendrites at baseline (iv), at 2 h (vi) and 24 h (ix) after incubation with mock immunodepleted (Aβ^+^) or Aβ‐immunodepleted (Aβ^−^) brain homogenate (ad BH) (v). Created in Biorender, J. Tulloch (2026), https://BioRender.com/406dt9h. (b) A typical example of longitudinal recording of the same dendrite before treatment (PRE), after 2 h incubation with ad BH (POST) and again the following day (24 h). Images are individual channels and the overlap of the neuron/tagBFP (blue), astrocyte/ECFP (cyan) and ptdSer/PsVue (magenta) channels. The neuron profile is outlined in semi‐transparent white in the last column panel, and a gaussian blur filter was applied to this column only. Scale bar 5 μm. (c) Relative changes in PsVue intensity at surviving and lost spines after 24 h Aβ^+^ or Aβ^−^ BH treatment. Here and in (c), large dots are animal averages, whereas small dots and violin plots are values from individual synapses. LMER details in text. (d) Relative changes in PsVue intensity at spines with proximal astrocytes (Y) or not (N). (e) Fraction of surviving spines after 24 h Aβ^+^ or Aβ^−^ BH treatment divided by astrocyte proximity. Violin plots are single dendrites, and points are individual animals.

To gain further insight onto the nature of the protective role of astrocytes we evaluated the amount of externalised phosphatidyl serine (ptdSer) on dendritic spines following the treatment with ad BH by means of fluorescent PsVue binding (Figure 5a,b). We found that, after 2 h incubation with Aβ^+^ BH, there was a significant increase in PsVue intensity compared with baseline. This was observed both at field level (Figure S10, LMER ΔPsVue/PsVue ~ Aβ*Sex + BH_batch + (1|animal, slice), Aβ *F*(1,18.34) = 4.00 *p* = 0.06, Sex *F*(1,5.03) = 0.81 *p* = 0.41, BH_batch *F*(1,5.07) = 0.42 *p* = 0.54, *n* = 24 slices from 13 animals) and at single spine level (LMER ΔPsVue/PsVue (Tukey transformed) ~ Aβ*Sex + BH_batch + (1|animal, slice), Sex *F*(1,8.18) = 1.38 *p* = 0.27, Aβ *F*(1,11.79) = 11.56 *p* = 0.0056 Aβ*Sex *F*(1,11.80) = 1.77 *p* = 0.21, BH_batch *F*(1,7.57) = 0.01 *p* = 0.92, *n* = 2959 spines from 13 animals. Two different BH preparations were used in this experiment (see Section 2.6). We observed that spines that would be lost at 24 h displayed higher exposure of ptdSer when treated with Aβ^+^ BH, but not Aβ^−^ BH (Figure 5c, LMER ΔPsVue/PsVue (Tukey transformed) ~ Aβ*Survival + Sex + BH_batch + (1|animal, slice), Survival *F*(1,2939.54) = 80.86 *p* < 0.0001, Aβ *F*(1,12.26) = 14.52 *p* = 0.0024, Aβ*Survival *F*(1,2939.07) = 39.82 *p* < 0.0001), Tukey post hoc comparison: (Aβ^−^| Survived—Eliminated) *z* = −1.06 *p* = 0.11 (Aβ^+^|Survived—Eliminated) *z* = −13.87 *p* < 0.0001, *n* = 2959 spines from 13 animals). Importantly, the increase in externalised ptdSer was lower in synapses with proximal astrocytes; this effect was specific for Aβ^+^ BH (Figure 5d, LMER ΔPsVue/PsVue (Tukey transformed) ~ Aβ*Astrocyte + Sex + BH_batch + (1|animal, slice), Astrocyte *F*(1,2940.88) = 33.32 *p* < 0.0001, Aβ *F*(1,12.50) = 12.67 *p* = 0.0037, Aβ*Astrocyte *F*(1,2937.75) = 17.88 *p* < 0.0001), Tukey post hoc comparison: (Aβ^−^| Astrocyte yes—no) *z* = 0.99 *p* = 0.32 (Aβ^+^| Astrocyte yes—no) *z* = 7.98 *p* < 0.0001, *n* = 2959 spines from 13 animals). Again, the proximity of astrocytes was protective against spine loss in an Aβ‐dependent manner (Figure 5e, LMER Survival ~ Aβ*Astrocyte + Sex + (1|animal, slice) Aβ *F*(1,20.82) = 8.97 *p* = 0.0069 Astrocyte *F*(1,22) = 10.81 *p* = 0.0034; Aβ*Astrocyte *F*(1,22) = 3.86 *p* = 0.062, Tukey post hoc comparison: (Aβ^−^| Astrocyte yes—no) *z* = −0.87 *p* = 0.39 (Aβ^+^|Astrocyte yes—no) *z* = −4.068 *p* = 0.0005, *n* = 24 dendrites from 13 animals).

### 3.7 Glutamate Removal Mediates Synapse Protection by Astrocytes

Our results so far indicate that Aβ increases the activity of neuronal synapses, and that at least a fraction of those displaying early changes is lost over 24 h. Although astrocytes do not have a net effect on synapse event frequency, their proximity was found to prevent synapse loss, which could potentially be mediated by lowering extrasynaptic glutamate levels. To determine whether astrocytes could protect synapses from loss through their role in clearing glutamate from the proximity of the spine, we repeated the experiment outlined in Figures 3 and 4 including TFB‐TBOA (hereafter, TBOA), a potent inhibitor of glutamate transporters GLAST and GLT1 (also known as EAAT1 and EAAT2), which are predominantly expressed in astrocytes (Pajarillo et al. 2019).

**FIGURE 6:**
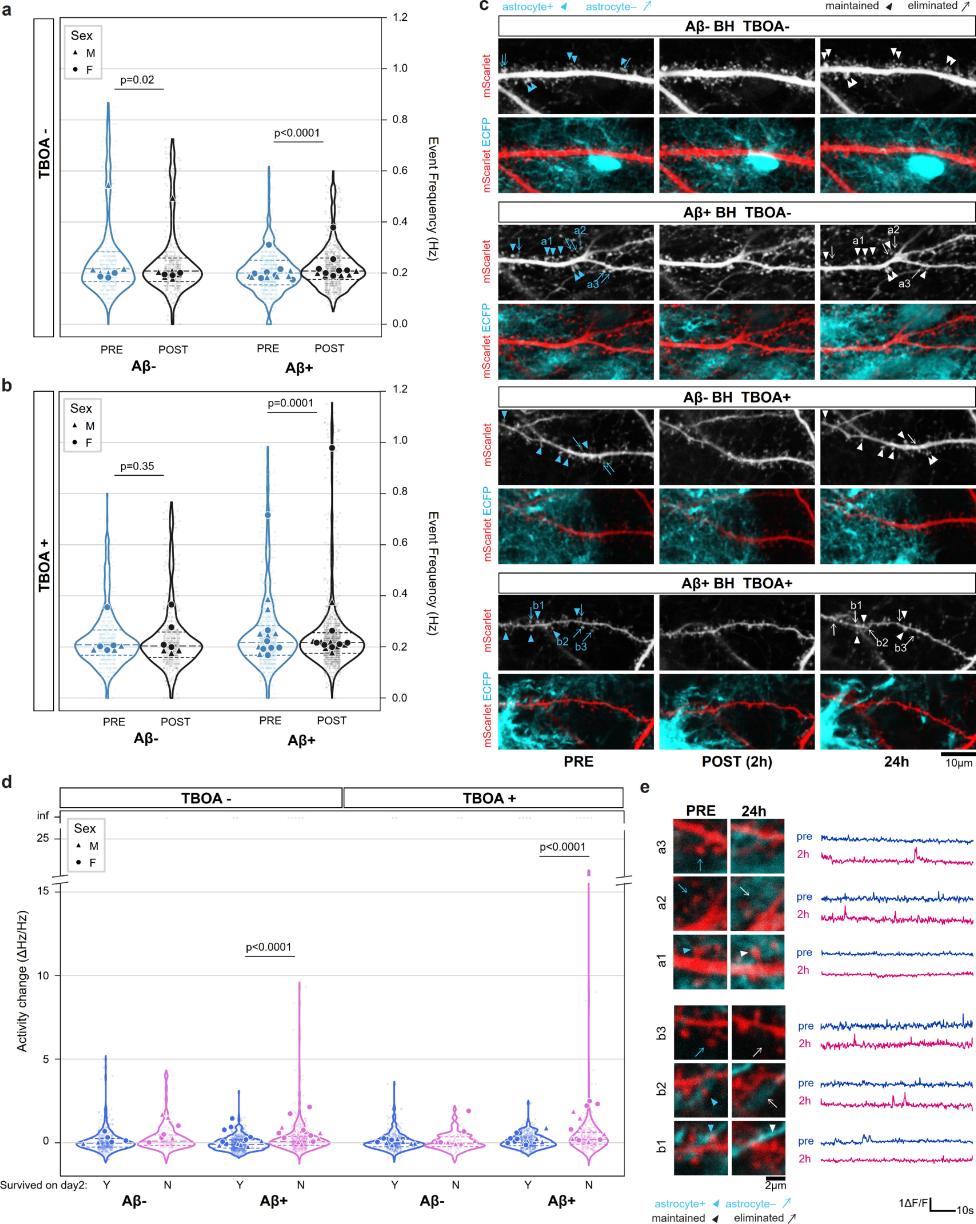
Effect of astrocyte glutamate transporter inhibition on synapse activity. (a, b) Event frequency for detected synapses before (PRE) and after treatment (POST) with Aβ^+^ or Aβ^−^ BH in presence of vehicle (a) or EAAT1/2 inhibitor TBOA (b). Small dots and violin plots are values from individual synapses, whereas large dots are animal averages. (d) Changes in event frequency after 2 h treatment with Aβ^+^ or Aβ^−^ BH, divided by synapses still present (Y) or lost (N) at 24 h. *Note:* A small number of infinite (inf) values occur where no events were detected in PRE recording and are displayed as “inf” on the y axis. LMER details are provided in text. (c) Examples of longitudinal recording of the same dendrite before treatment (PRE), after 2 h incubation with ad BH (POST) and again the following day (24 h), under various conditions indicated above. Images are the neuron/mScarlet (grayscale) channel and its overlap (red) with astrocyte/ECFP (cyan). Full images and individual channels are presented in Figure S11. Scale bar 10 μm. (d) Relative changes in synaptic frequency for surviving and lost spines after 24 h Aβ^+^ or Aβ^−^ BH treatment, with either vehicle or TBOA. LMER details in text. (e) Representative ΔF/F traces of GCaMP fluorescence over 60 s before (pre) and after treatment (post) for synapses a1‐3 (Aβ^+^, vehicle) and b1‐3 (Aβ^+,^ TBOA) in panel (c). Scale bar 1 ΔF/F, 10 s.

We found a significant interaction between Aβ BH, TBOA and treatment time in the frequency of synaptic events (Figures 6a–c and S6). LMER Event rate ~ Treatment time (PRE/POST treatment)*Aβ*TBOA + Sex+ (1|animal, slice); Aβ*Treatment Time*TBOA *F*(1,5839) = 29.5 *p* = 5.6 e‐08, Sex *F*(1,17.6) = 1.38, *p* = 0.26. Tukey post hoc comparisons: (Aβ^−^ TBOA^−^| POST—PRE) z ratio = −2.364 *p* = 0.018, (Aβ^−^ TBOA^+^| POST—PRE) z ratio = 0.932 *p* = 0.351, (Aβ^+^ TBOA^−^| POST—PRE) z ratio = 4.525 *p* < 0.0001, (Aβ^+^ TBOA^+^|POST—PRE) z ratio = 3.877 *p* = 0.0001, *n* = 2952 spines from 26 animals/60 slices). Lost spines at 24 h were more likely to show early increases in their activity at 2 h (Figures 6c–e and S11). LMER ΔHz/Hz (Tuckey transformed) ~ Aβ*TBOA*Survival + Sex + (1|animal, slice); Aβ *F*(1,67.37) = 1.40 *p* = 0.24, Survival *F*(1,2903.9) = 47.038 *p* < 0.0001, Aβ*Survival *F*(1,2906) = 13.14 *p* = 0.0003, Aβ*Survival*TBOA *F*(1,2903) = 5.01 *p* = 0.025, Sex *F*(1,20.18) = 7.27, *p* = 0.014), both with and without inhibitors of glutamate transporters (Tuckey post hoc comparisons: (Aβ^−^ TBOA^−^| Survived—Eliminated) z ratio = −0.087 *p* = 0.93, (Aβ^−^ TBOA^+^| Survived—Eliminated) z ratio = −2.78 *p* = 0.0068, (Aβ^+^ TBOA^−^| Survived—Eliminated) z ratio = −7.139 *p* < 0.0001, (Aβ^+^ TBOA^+^|Survived—Eliminated) z ratio = −7.067 *p* < 0.0001, *n* = 2918 spines from 26 animals/60 slices). Again, we found a non‐significant effect of astrocytic proximity on early activity, with a minor but significant interaction between TBOA and Astrocyte presence (Figure S7a, LMER ΔHz/Hz (Tuckey transformed) ~ Aβ*TBOA*Astrocyte + Sex + (1|animal, slice); Astrocyte *F*(1, 2491.57) = 0.64 *p* = 0.42, TBOA*Astrocyte *F*(1, 2598.37) = 6.26 *p* = 0.0124).

**FIGURE 7:**
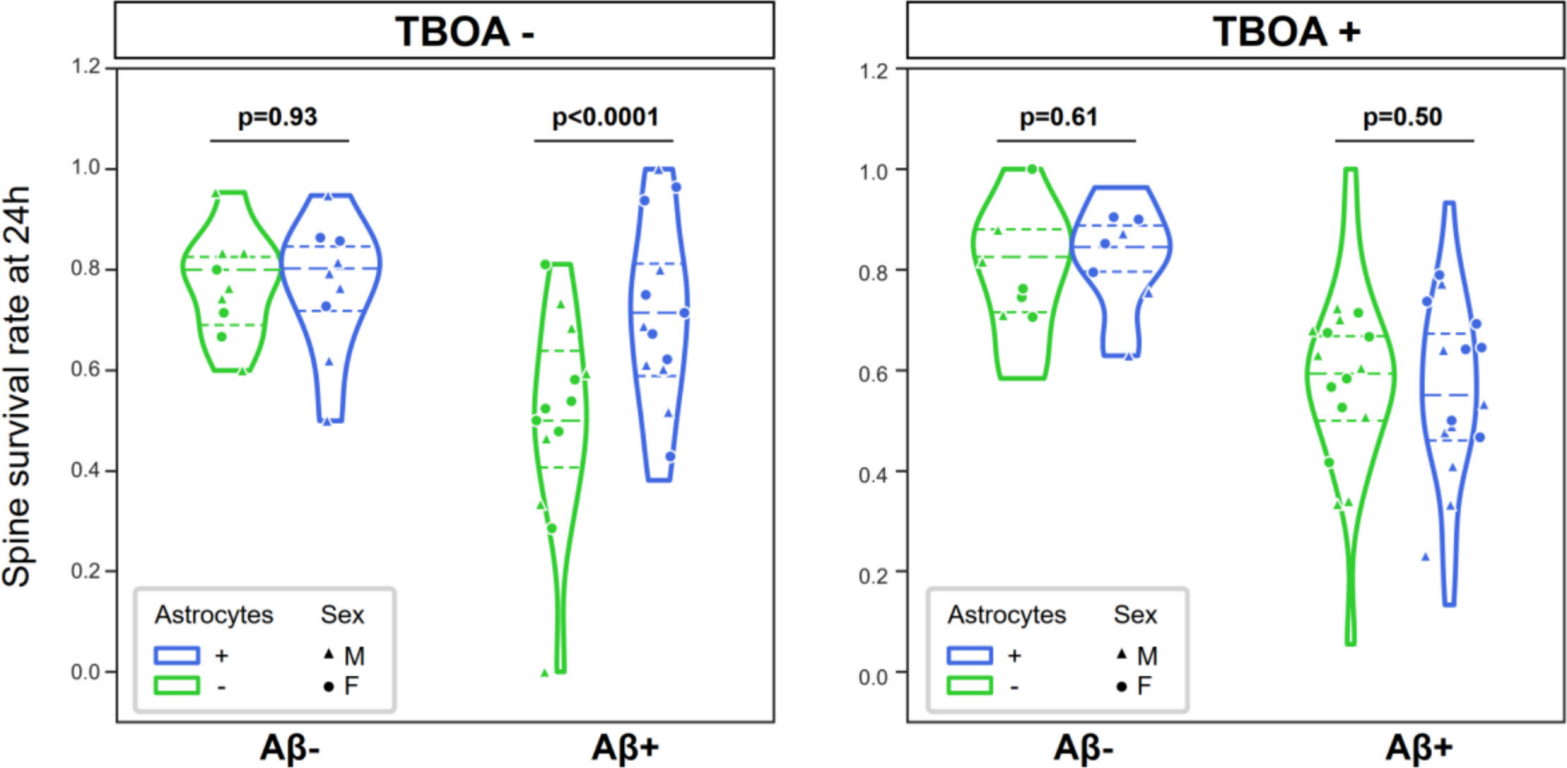
Effect of astrocyte glutamate transporter inhibition on spine survival. Fraction of surviving spines after 24 h Aβ^+^ or Aβ^−^ BH treatment divided by astrocyte proximity and TBOA inclusion in medium. Violin plots are individual dendrites, and dots are animal averages. LMER details in text.

We then turned our attention to the astrocyte effect on spine survival. In vehicle‐treated slices, we confirmed the observed protective role of astrocytes (Figure 7). Including the TBOA astrocytic glutamate transporter inhibitor prevented this effect: in slices treated with Aβ^+^ BH and TBOA, spine with and without astrocytes in their proximity had similar survival rates (Figures 6c and S12, LMER Survival ~ Aβ*Astrocyte*TBOA + Sex + (1|animal, slice) Aβ *F*(1,55.51) = 26.22 *p* < 0.0001, Astrocyte *F*(1,56.05) = 4.52 *p* = 0.037, TBOA *F*(1,55.53) = 0.004 *p* = 0.94, Sex *F*(1,55.03) = 1.40 *p* = 0.24, Aβ*Astrocyte *F*(1,56.05) = 2.71 *p* = 0.10, TBOA*Astrocyte *F*(1,56.05) = 4.12 *p* = 0.04, TBOA* Aβ*Astrocyte *F*(1,56.05) = 7.45 *p* = 0.0083; Tukey post hoc comparison: (Aβ^−^ TBOA^−^| Astrocyte yes—no) z ratio = −0.102 *p* = 0.9193, (Aβ^−^ TBOA^+^| Astrocyte yes—no) z ratio = 0.519 *p* = 0.6056, (Aβ^+^ TBOA^−^| Astrocyte yes—no) z ratio = 4.576 *p* < 0.0001, (Aβ^+^ TBOA^+^|Astrocyte yes—no) z ratio = −0.673 *p* = 0.5040, *n* = 60 dendrites from 26 animals).

## 4 Discussion

Our results in mouse hippocampal organotypic slices demonstrate that challenging neurons with BH from ad patients containing soluble Aβ forms (King et al. 2023; Sideris et al. 2021) can induce an increase of synaptic activity and significant synapse loss. Furthermore, lost synapses were associated with increases in synaptic event rate in response to Aβ^+^ BH (Figure 3). This suggests a link between Aβ‐induced increase in activity and synapse removal. Our results are consistent with studies linking neuronal and dendritic hyperactivity to Aβ species and plaque proximity (Palop et al. 2007; Busche et al. 2008; Kuchibhotla et al. 2008; Arbel‐Ornath et al. 2017; Schultz et al. 2017; Henstridge, Tzioras, and Paolicelli 2019). Although removing Aβ has been shown to be protective against synapse loss in mouse models of amyloid deposition (Spires‐Jones et al. 2009), chronic pharmacological synaptic inhibition decreases plaque load but does not prevent synapse loss or memory impairments in mice, implying reducing activity alone is not sufficient to prevent synapse loss (Tampellini et al. 2010). This suggests that the observed increase in synaptic activity after Aβ^+^ BH challenge has distinct features from physiological activity. For instance, pathological activation could be accompanied by the exposure of signalling molecules like phosphatidyl serine or complement proteins like C1q or C3 (Hong et al. 2016; Rueda‐Carrasco et al. 2023).

**FIGURE 8:**
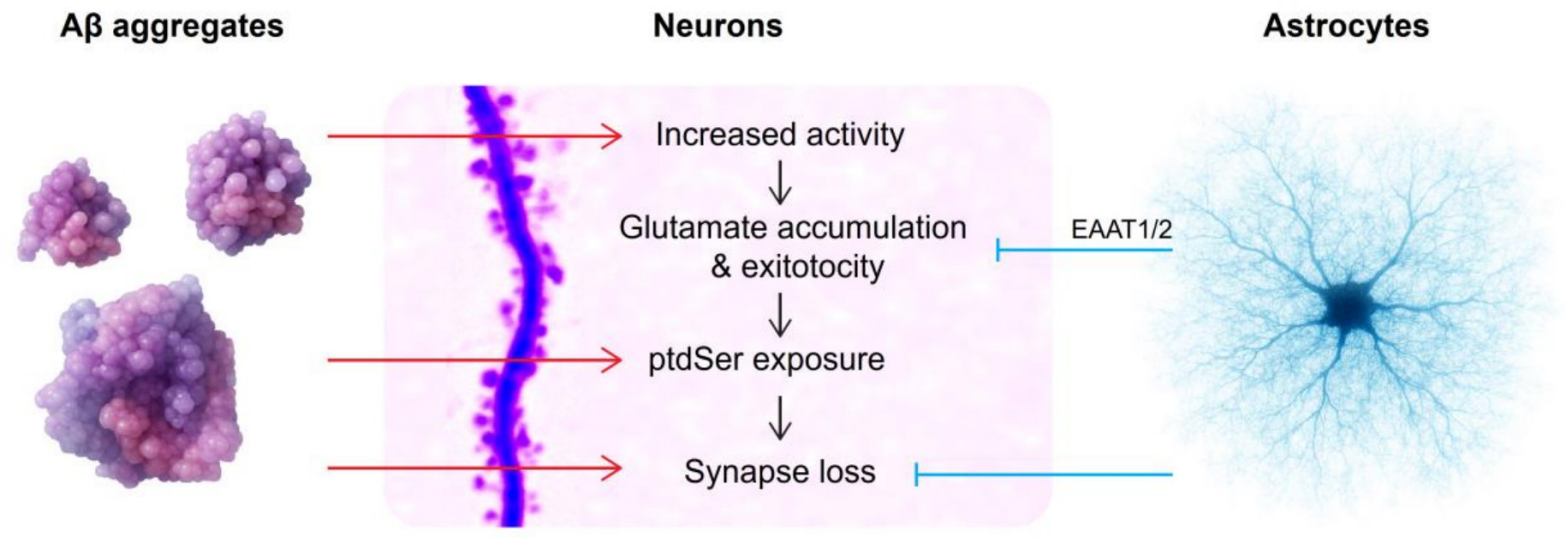
Summary of findings: Astrocytes protect synapses from Aβ‐induced glutamate toxicity. Aβ aggregates present in Aβ^+^ BH are responsive for increased synaptic activity, ptdSer exposure and synapse loss. We propose that glutamate toxicity due to hyperactivity can, at least in part, mediate the observed synapse loss. Astrocytes were found to prevent synapse loss and reduce ptdSer exposure in their proximity, but had no effect on activity reduction, suggesting that their effect lies downstream of changes in activity and involves glutamate removal via astrocytic glutamate transporters. Cells and molecules in the figure are artistic representations generated in part with Microsoft Copilot and are not scientific images.

In our experiments, we found astrocyte proximity to synapses to be a strong factor predicting synapse survival at 24 h (Figures 4 and 5). Because our experiment models an early, relatively mild insult by Aβ toxic species, this suggests that astrocytes can actively counteract Aβ effects. Our results indicate that synapses close to astrocytes display a reduced exposure of phosphatidyl serine (ptdSer) after challenging the cultures with Aβ^+^ BH. PtdSer has been identified as a potent signalling molecule and in dissociated cultures microglia can phagocytose synapses expressing externalised ptdSer (Rueda‐Carrasco et al. 2023). Together, our data suggest that astrocytes have a protective role against synapse loss. It remains to be determined whether this protective action is due to direct removal of Aβ deposits from the spine surface or the perisynaptic space (Wyss‐Coray et al. 2003; Rolyan et al. 2011), or if there are downstream effects that counteract Aβ toxicity. For instance, astrocytes are known to contribute to synapse physiology by sensing synaptic activity, regulating glutamate levels and releasing gliotransmitters (Mahmoud et al. 2019; Noriega‐Prieto and Araque 2021). Even though astrocyte proximity is not sufficient to prevent synaptic hyperactivity, blocking glutamate transporters prevented the protective effect. This could be due, at least in part, to removal of excess perisynaptic glutamate, that is associated with excitotoxicity (Figure 8) (Hardingham and Bading 2010). Notably, others have reported that astrocytes can prune synapses during development or after sustained increase in activity, with a net homeostatic effect (Chung et al. 2013; Lee et al. 2021). Furthermore, in advanced stages of ad, astrocytes from human cases engulf significantly more synapses than control samples (Tzioras, Daniels, et al. 2023) and synapses containing p‐tau Ser356 are five times more likely to be ingested by an astrocyte than synapses lacking tau (Taylor et al. 2024). This suggests that with ad progression astrocytes shift towards a more aggressive phenotype or become incapable of fulfilling their protective function (Hulshof et al. 2022). Indeed, it has been reported that even in more advanced stages, reactive astrocytes express a combination of genes with both neuroprotective and neurotoxic effects (Jiwaji et al. 2022; Serrano‐Pozo et al. 2024). It is also possible then that ad synaptic material phagocytosed by astrocytes are dysfunctional synapses or represent lingering debris resulting from prior synapse degeneration (Hulshof et al. 2022).

Our data are therefore consistent with a pro‐homeostatic role of astrocytes and suggest that astrocytes may exert a beneficial role contrasting the deleterious effects of Aβ species in the earlier phases of ad. In vivo experiments at various stages of Aβ pathology will address whether this also happens in the context of physiologically relevant synaptic activity. One limitation of our study is the use of an ex vivo model where, notwithstanding the advantages it offers (Durrant 2020; McGeachan et al. 2025), synaptic transmission is the result of spontaneous, recurrent activity with little physiological meaning. A second limitation is the use of GECIs to estimate synaptic activity. The identification of transmission events with calcium indicators is notoriously difficult due to the signal‐to‐noise ratio of imaging fast calcium transients in small, optically underresolved structures like dendritic spines (Yasuda et al. 2004; Ishikawa et al. 2020), but offers the advantage over electrophysiology to determine the activity rate of each synapse. Although calcium imaging is generally considered to offer a good measure of neuronal activity, it must be remembered that the relationship between action potentials and calcium events is not one‐on‐one (Huang et al. 2021), even though newer GECIs tend to perform better than previous generations (Dana et al. 2019; Zhang et al. 2023). Voltage indicators or fluorescent glutamate sensors could be employed to obtain complementary information (Marvin et al. 2018).

Our study has the advantage of using live, longitudinal imaging to measure changes in neuronal activity and spine loss. Furthermore, it employs ECFP as a marker of astrocytic volume, allowing the visualisation of fine processes in proximity to synapses (Figure 2). Thus, some of the discrepancies might be due to the use of live imaging instead of staining for astrocytic markers such as GFAP. It must be noted, however, that our identification of synapses is based on the presence of spines. Although virtually all spines constitute postsynaptic terminals (Nimchinsky et al. 2002; Arellano 2007), this might introduce some biases in the quantification of the results. In particular, astrocytes have been reported to preferentially internalise presynaptic boutons (Lee et al. 2021). However, we have observed internalisation of both presynaptic terminals and postsynaptic densities by astrocytes in human ad brain (Tzioras, Daniels, et al. 2023). In living human brain slices from tissue resected during neurosurgery, we observed ad BH causes a marked reduction of presynaptic puncta densities, whereas no significant effect was measured for PSD95 (McGeachan et al. 2025). Hence, astrocyte phagocytosis of synapses could act preferentially on presynaptic terminals. Although we do not think that this factor alone explains our data, it needs to be taken into consideration that our definition of synapse association with astrocytes is based on astrocyte‐postsynapse proximity. A repetition of the experiment observing presynaptic terminals instead of spines could help elucidate this aspect, even though the relationship between axonal boutons and presynapses is less clear, thus requiring the live labelling of a presynaptic protein. Furthermore, intrinsic limitations in fluorescence microscopy prevent us from distinguishing astrocyte proximity from physical contact. Astrocyte processes can display a complex and ramified network around groups of synapses in physiological and pathological conditions (Benoit et al. 2025); indeed, our data suggest that the pro‐survival role of astrocyte proximity is, at least in part, due to glutamate removal from the peri‐synaptic space, demonstrating a functional role for the observed spine‐astrocyte interaction.

Last, if astrocytes have a protective role in our experiment, it is unclear whether the observed loss of synapses is due to a cell‐autonomous mechanism activated by neurons or is mediated by other cell types. An obvious candidate would be microglia that have been reported to engulf hyperactive synapses in dissociated neuron cultures treated with synthetic Aβ (Rueda‐Carrasco et al. 2023). Mouse lines expressing fluorescent proteins that could be used in parallel experiments in combination with green or red GECIs have been recently reported (Kaiser and Feng 2019; Ruan et al. 2020).

In conclusion, our results demonstrate that soluble Aβ induces synaptic hyperactivity and loss and that astrocyte proximity has an overall protective role against an acute soluble Aβ insult. Future studies will elucidate how this role evolves with prolonged or increased challenge with toxic species. Understanding how the role of astrocytes dynamically changes in the course of AD and what factors are driving these changes will offer critical insight onto how to preserve or inhibit their activity when designing therapeutical approaches to preserve synaptic integrity.

## Author Contributions

**Francesco Gobbo:** investigation, methodology, software, formal analysis, data curation, writing – original draft, writing – review and editing, visualization, project administration. **Declan King:** methodology, writing – review and editing. **Jane Tulloch:** resources. **Davide Gobbo:** resources, writing – review and editing. **Calum Bonthron:** investigation. **Soraya Meftah:** investigation. **Caleb Stoddart‐Campbelton:** investigation. **Arisa Tamura:** investigation. **Jamie Rose:** investigation. **Colin Smith:** resources, supervision. **Claire Durrant:** methodology, writing – review and editing, supervision. **Tara L. Spires‐Jones:** conceptualization, methodology, software, formal analysis, data curation, writing – review and editing, supervision, funding acquisition.

## Funding

This work is supported by the UK Dementia Research Institute (Award Number UK DRI‐4204) to Tara L. Spires‐Jones through UK DRI Ltd, which is principally funded by the UK Medical Research Council. Durrant receives funding from Race Against Dementia (ARUK‐RADF‐2019a‐001), The James Dyson Foundation and the Alzheimer's Society (AS‐PG‐21‐006).

## Conflicts of Interest

T.L.S‐J. has no direct conflicts with this study but has received payments for consulting, scientific talks or collaborative research over the past 10 years from AbbVie, Sanofi, Merck, Scottish Brain Sciences, Jay Therapeutics, Cognition Therapeutics, Ono, Novo Nordisk and Eisai, and directs a company, Spires‐Jones Neuroscience Ltd, to act as a consultant. She is also a charity trustee for the British Neuroscience Association and the Guarantors of Brain and serves as a scientific advisor to several charities and non‐profit institutions.

## Supporting information


**Figure S1:** Comparison of genetically encoded calcium indicators (GECIs). (a) Representative dendrites expressing the three GECIs. Scale bar, 5 μm. (b) Single synapse temporal fluorescence traces. Top panels (green): ΔF/F traces expressed as z‐scores. Bottom panels: OASIS deconvolved traces (blue) and detected events (red dots). (c) Normalised fluorescence profiles for the three GECIs (mean ± SEM). (d) Bleach rate quantified for the three GECIs (see Methods). (e) Single event temporal profile of fluorescence in ΔF/F units, z‐scored normalised (top panels) or as absolute units (bottom panels). Light traces represent individual events profiles, and the average is presented as dashed line. (f) Quantification of event amplitude in ΔF/F units measured at peak for the three GECIs.
**Figure S2:** Processing of synaptic calcium traces. (a) Mean grey values from synaptic ROIs were processed by subtracting the baseline background with a polynomial fit (black line) and denoised with four consecutive iterations of the Okada filter. Displayed are successive iterations for a typical synaptic trace. (b) The resulting traces are then converted in ΔF/F units.
**Figure S3:** Specificity of ECFP expression. Histological staining of hippocampal slices from GCFD animals showing no overlap between ECFP (cyan in merge) and neuronal marker NeuN (red in merge) staining. Scale bar, 50 μm.
**Figure S4:** ECFP expression in MOHSC. Histological staining of mouse organotypic hippocampal slices from GCFD animals showing strong overlap between ECFP (cyan) and astrocytic marker GFAP (red) staining. Representative images from two different animals. Scale bar, 25 μm. On the right, quantification of ECFP/GFAP overlap in slices from *n* = 4 animals.
**Figure S5:** Activity change in wild‐type slices. (a) We confirmed the observed increase in activity change in activity with Aβ+ brain homogenate. Small points are individual synapses, whereas large dots are animal averages. LMER *p* < 0.001 (see text). (b) Synapse loss was comparable in neurons from wild‐type slices. LMER Survival_probability ~ Genotype + (1|animal) *z* = 0.84 *p* = 0.404, *n* = 33 animals. Note that synapse loss for GCFD slices was calculated as loss per dendrite without dividing by astrocyte contact to make data comparable with values from wild‐type animals. Points are individual animals.
**Figure S6:** Activity change by astrocytes. ΔF/F change in activity for individual synapses after 2 h treatment with Aβ+ or Aβ− brain homogenate divided by whether they were contacted by astrocytes or not.
**Figure S7:** Activity change at 24 h. (a) Event frequency for a subset of synapses identified both at baseline and at 24 h; before (PRE), after treatment (POST) and at 24 h (24 h) Aβ+ or Aβ− BH. Small dots and violin plots are values from individual synapses, whereas large dots are animal averages. (c) Changes in event frequency after 24 h treatment with Aβ+ or Aβ− BH. Large dots are animal averages, whereas small dots and violin plots are values from individual synapses. (c) Same as (b) divided by astrocyte proximity.
**Figure S8:** Preferred survival of synapses in proximity of astrocyte processes. Additional example of a dendrite treated with Aβ+ BH showing preferential loss of synapses devoid of astrocyte processes in their vicinity. Scale bar, 10 μm.
**Figure S9:** Aβ+ BH does not induce a significant loss of astrocytes. (a) Quantification of astrocytes per field of view after 24 h Aβ− or Aβ+ BH treatment. (b) Representative fields with astrocytes in cyan. The quantification was performed from large field of view acquired in the PsVue experiment. Neurons in images express TagBFP. Scale bar, 20 μm.
**Figure S10:** Increased exposure of phosphatidyl serine after BH challenge. ΔPsVue/PsVue change for individual fields of view after 2 h treatment with Aβ+ or Aβ− brain homogenate.
**Figure S11:** Full panels of images cropped in Figure 5.
**Figure S12:** Changes in activity by proximity of astrocytes. (a) ΔHz/Hz for synapses treated with Aβ− or Aβ+ BH, with either vehicle or TBOA. (b) Overall synapse survival at 24 h after treatment with Aβ− or Aβ+ BH, with either vehicle or TBOA.

## Data Availability

The code used in the paper is available at https://github.com/francesco‐gobbo/Astrocyte‐proximity‐protects‐synapses‐in‐AD/tree/main. The data used in this paper are available at Edinburgh DataShare: https://doi.org/10.7488/ds/8077 (within the collection https://datashare.ed.ac.uk/handle/10283/3076). Original images are available from the corresponding author upon request.
