# Supplementary material for: Astrocyte Proximity Protects Synapses From Human Amyloid‐Beta Induced Degeneration in a Mouse Ex Vivo Model of Early Alzheimer's Disease
Source: Eur J Neurosci. 2026 Mar 28;63(7):e70480. doi: 10.1111/ejn.70480 (PMC13032744; doi:10.1111/ejn.70480)
Supplement: Supplementary file 1 — Figure S1: Comparison of genetically encoded calcium indicators (GECIs). (a) Representative dendrites expressing the three GECIs. Scale bar, 5 μm. (b) Single synapse temporal fluorescence traces. Top panels (green): ΔF/F traces expressed as z‐scores. Bottom panels: OASIS deconvolved traces (blue) and detected events (red dots). (c) Normalised fluorescence profiles for the three GECIs (mean ± SEM). (d) Bleach rate quantified for the three GECIs (see Methods). (e) Single event temporal profile of fluorescence in ΔF/F units, z‐scored normalised (top panels) or as absolute units (bottom panels). Light traces represent individual events profiles, and the average is presented as dashed line. (f) Quantification of event amplitude in ΔF/F units measured at peak for the three GECIs. Figure S2: Processing of synaptic calcium traces. (a) Mean grey values from synaptic ROIs were processed by subtracting the baseline background with a polynomial fit (black line) and denoised with four consecutive iterations of the Okada filter. Displayed are successive iterations for a typical synaptic trace. (b) The resulting traces are then converted in ΔF/F units. Figure S3: Specificity of ECFP expression. Histological staining of hippocampal slices from GCFD animals showing no overlap between ECFP (cyan in merge) and neuronal marker NeuN (red in merge) staining. Scale bar, 50 μm. Figure S4: ECFP expression in MOHSC. Histological staining of mouse organotypic hippocampal slices from GCFD animals showing strong overlap between ECFP (cyan) and astrocytic marker GFAP (red) staining. Representative images from two different animals. Scale bar, 25 μm. On the right, quantification of ECFP/GFAP overlap in slices from n = 4 animals. Figure S5: Activity change in wild‐type slices. (a) We confirmed the observed increase in activity change in activity with Aβ+ brain homogenate. Small points are individual synapses, whereas large dots are animal averages. LMER p < 0.001 (see text). (b) Synapse loss was c [file EJN-63-0-s001.pdf]

Astrocyte proximity protects synapses from human amyloid-beta induced degeneration in a mouse in vitro model of early Alzheimer's disease

Francesco Gobbo[1], Declan King[1], Jane Tulloch[1], Davide Gobbo[2], Calum Bonthron[1], Soraya Meftah[1], Caleb Stoddart-Campbelton[3], Arisa Tamura[4], Jamie Rose[1], Colin Smith[1], Claire Durrant[1], Tara Spires-Jones[1]

[1]INCR and UK Dementia Research Institute, The University of Edinburgh, 1 George Square, EH8 9JZ, Edinburgh, United Kingdom.

[2]Department of Molecular Physiology, Centre for Integrative Physiology and Molecular Medicine (CIPMM), University of Saarland, Building 48, 66421, Homburg, Germany,

[3]Undergrad course in General Neuroscience, The University of Edinburgh, 1 George Square, EH8 9JZ, Edinburgh, United Kingdom.

[4]Department of Biological Sciences, Tokyo Metropolitan University, Tokyo, Japan.

Supplementary Material

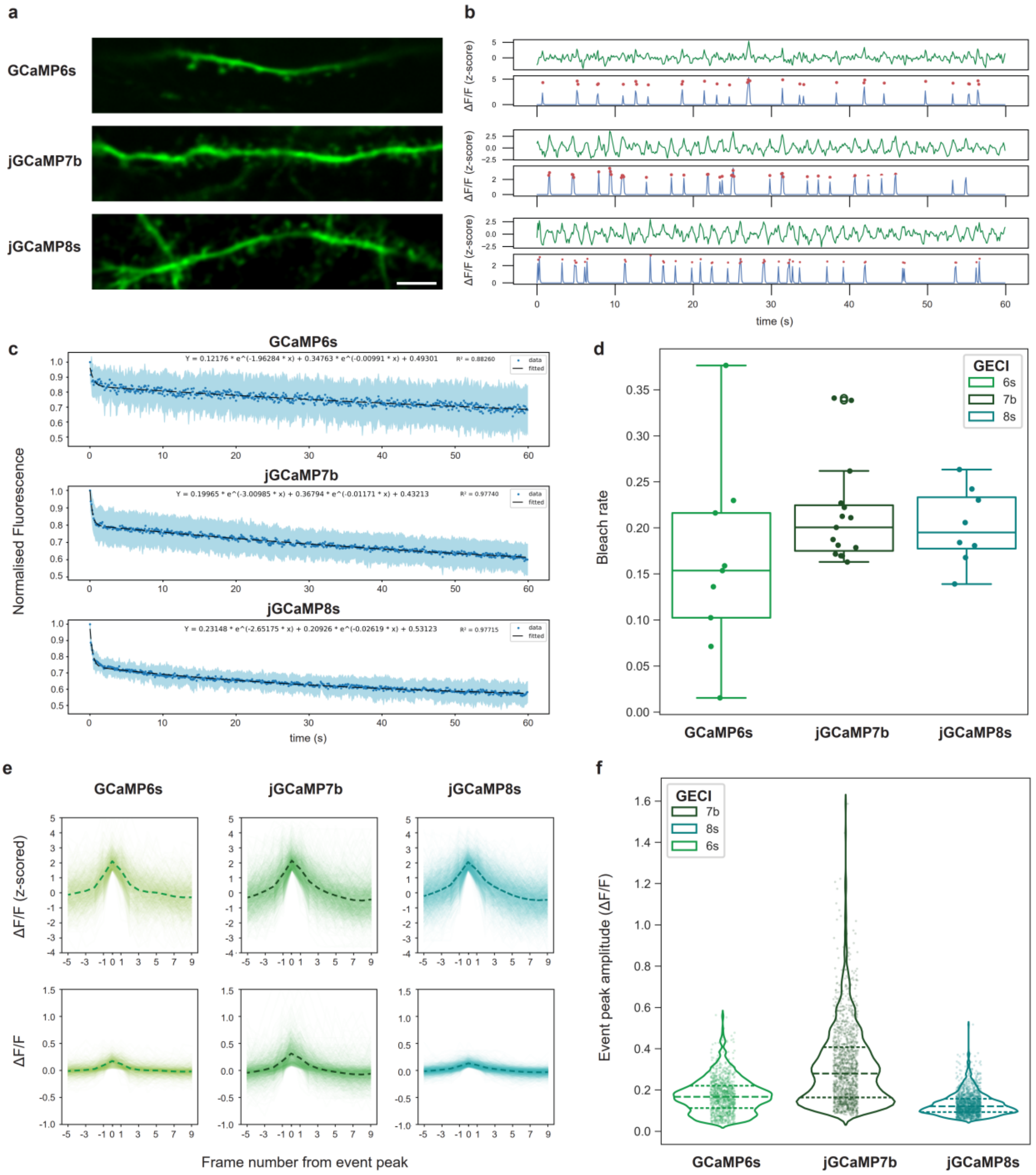

Figure S1 Comparison of genetically encoded calcium indicators (GECIs) a) Representative dendrites expressing the three GECIs. Scale bar, 5 µm. **b**) Single synapse temporal fluorescence traces. Top panels (green): ΔF/F traces expressed as z-scores. Bottom panels: OASIS deconvolved traces (blue) and detected events (red dots). **c**) Normalised fluorescence profiles for the three GECIs (mean±sem). **d**) Bleach rate quantified for the three GECIs (see Methods). **e**) Single event temporal profile of fluorescence in ΔF/F units, z-scored normalised (top panels) or as absolute units (bottom panels). Light traces represent individual events profiles,

and the average is presented as dashed line. **f**) Quantification of event amplitude in ΔF/F units measured at peak for the three GECIs.

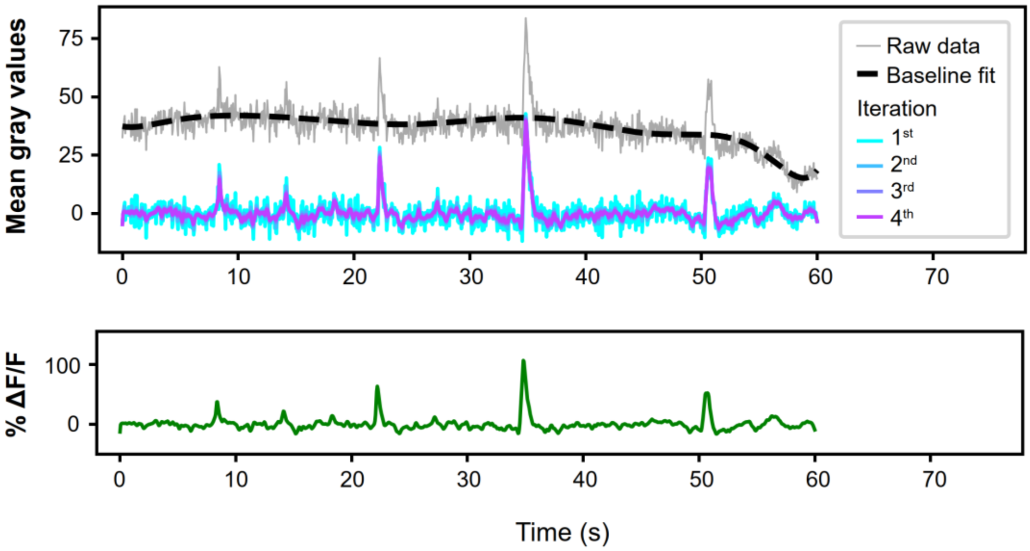

Figure S2 Processing of synaptic calcium traces. a) Mean gray values from synaptic ROIs were processed by subtracting the baseline background with a polynomial fit (black line) and denoised with four consecutive iterations of the Okada filter. Displayed are successive iterations for a typical synaptic trace. **b**) The resulting traces are then converted in ΔF/F units.

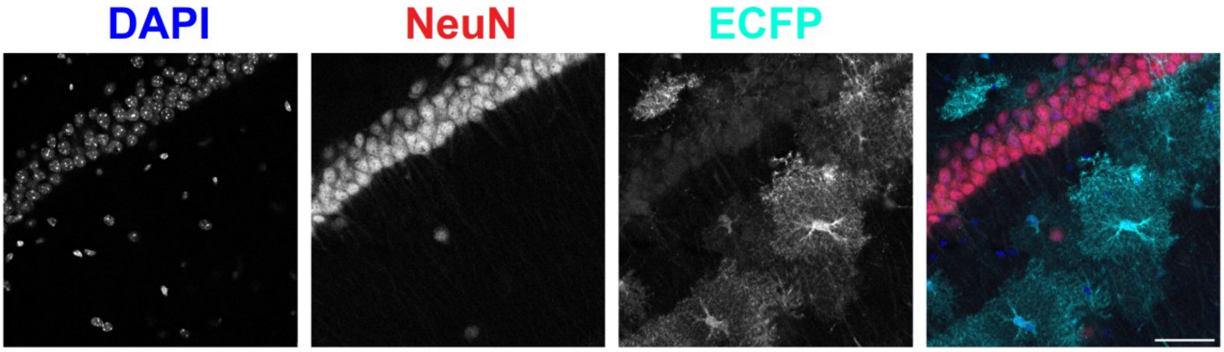

Figure S3 Specificity of ECFP expression. Histological staining of hippocampal slices from GCFD animals showing no overlap between ECFP (cyan in merge) and neuronal marker NeuN (red in merge) staining. Scale bar, 50 μm.

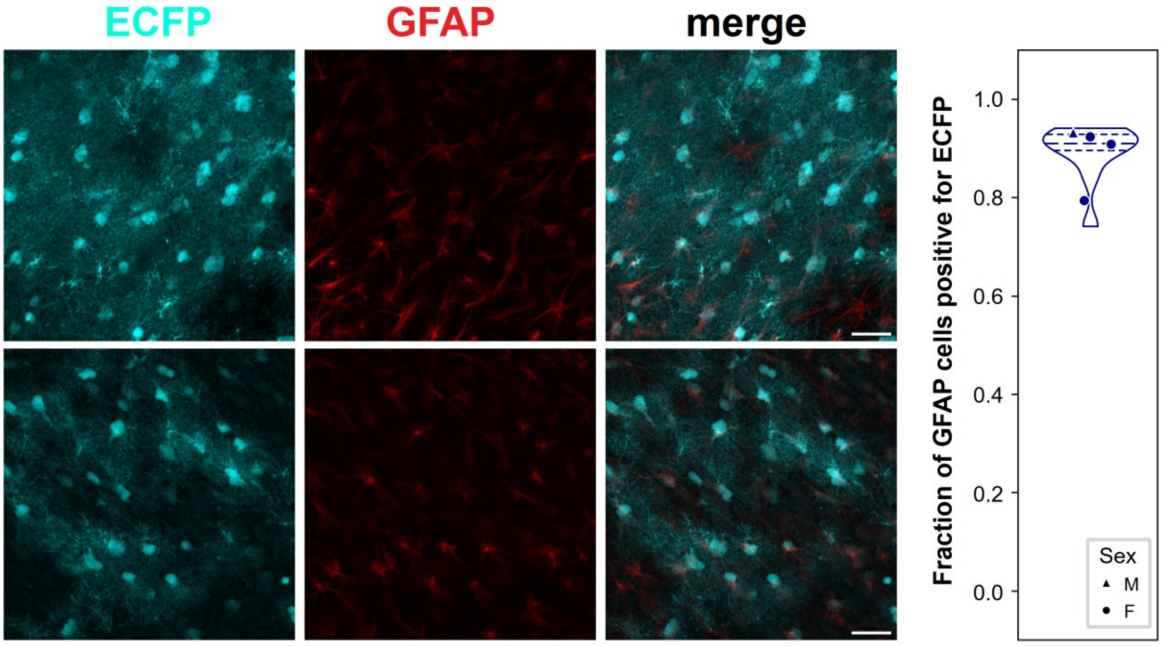

Figure S4 ECFP expression in MOHSC. Histological staining of mouse organotypic hippocampal slices from GCFD animals showing strong overlap between ECFP (cyan) and astrocytic marker GFAP (red) staining. Representative images from two different animals. Scale bar, 25 µm. On the right, quantification of ECFP/GFAP overlap in slices from n=4 animals.

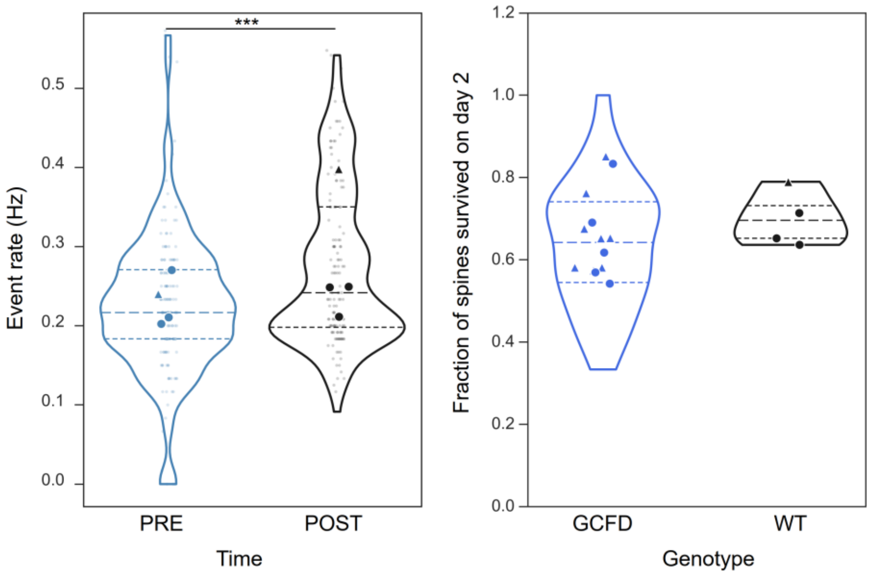

Figure S5 Activity change in wild-type slices. a) We confirmed the observed increase in activity change in activity with Aβ+ brain homogenate. Small points are individual synapses, while large dots are animal averages. LMER $p<0.001$ (see text). **b**) Synapse loss was comparable in neurons from wild-type slices. LMER Survival_probability ~ Genotype + (1|animal) $z=0.84$ $p=0.404$, $n=33$ animals. Note that synapse loss for GCFD slices was calculated as loss per dendrite without dividing by astrocyte contact to make data comparable with values from wild-type animals. Points are individual animals.

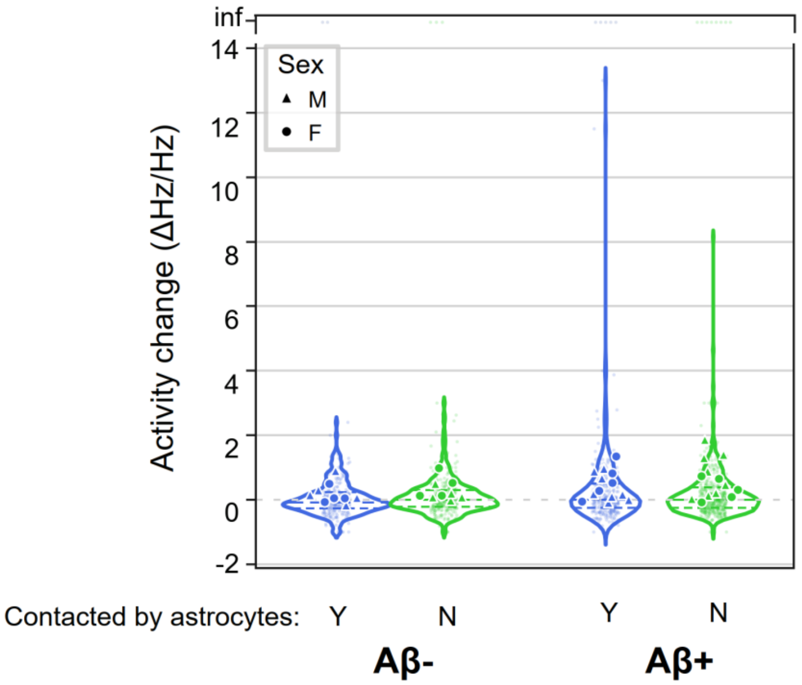

Figure S6 Activity change by astrocytes. ΔF/F change in activity for individual synapses after 2h treatment with Aβ+ or Aβ- brain homogenate divided by whether they were contacted by astrocytes or not.

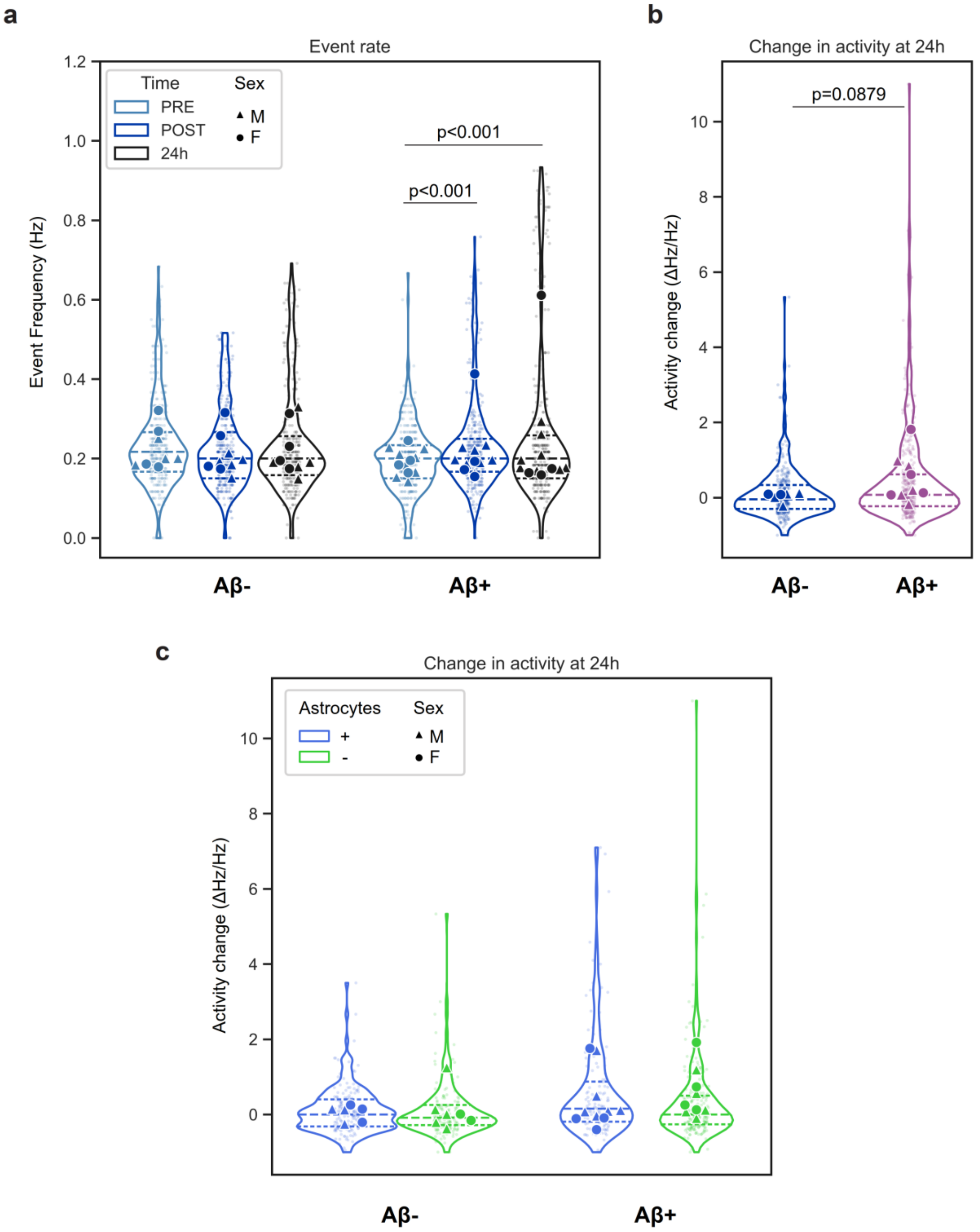

Figure S7 Activity change at 24h. a) Event frequency for a subset of synapses identified both at baseline and at 24h; before (PRE), after treatment (POST), and at 24h (24h) Aβ+ or Aβ- BH. Small dots and violin plots are values from individual synapses, while large dots are animal averages. c) Changes in event frequency after 24h treatment with Aβ+ or Aβ- BH. Large dots are animal averages, while

small dots and violin plots are values from individual synapses. c) Same as b) divided by astrocyte proximity.

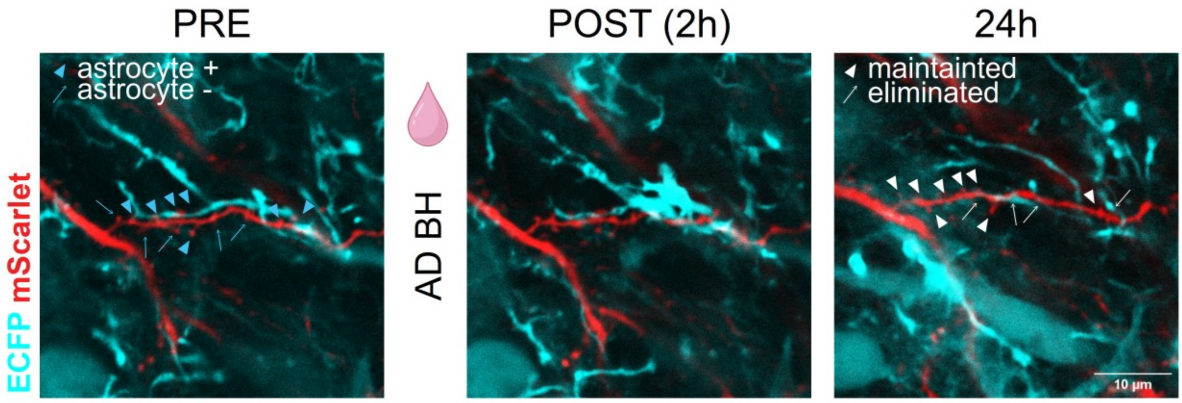

Figure S8 Preferred survival of synapses in proximity of astrocyte processes. Additional example of a dendrite treated with Aβ+ BH showing preferential loss of synapses devoid of astrocyte processes in their vicinity. Scale bar, 10 µm.

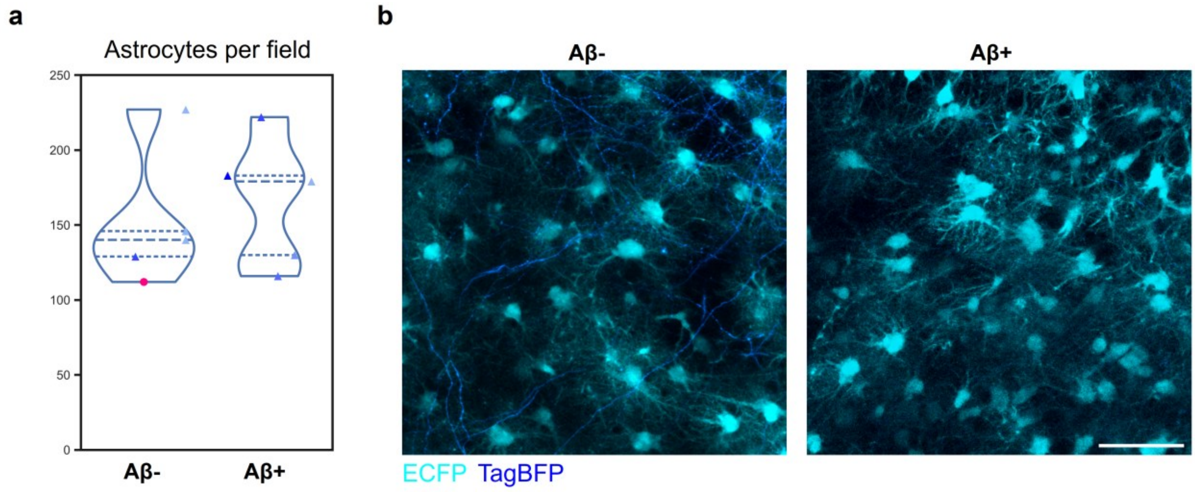

Figure S9 Aβ+ BH does not induce a significant loss of astrocytes. a) Quantification of astrocytes per field of view after 24h Aβ- or Aβ+ BH treatment. b) Representative fields with astrocytes in cyan. The quantification was performed from large field of view acquired in the PsVue experiment. Neurons in images express TagBFP. Scale bar, 20 μm.

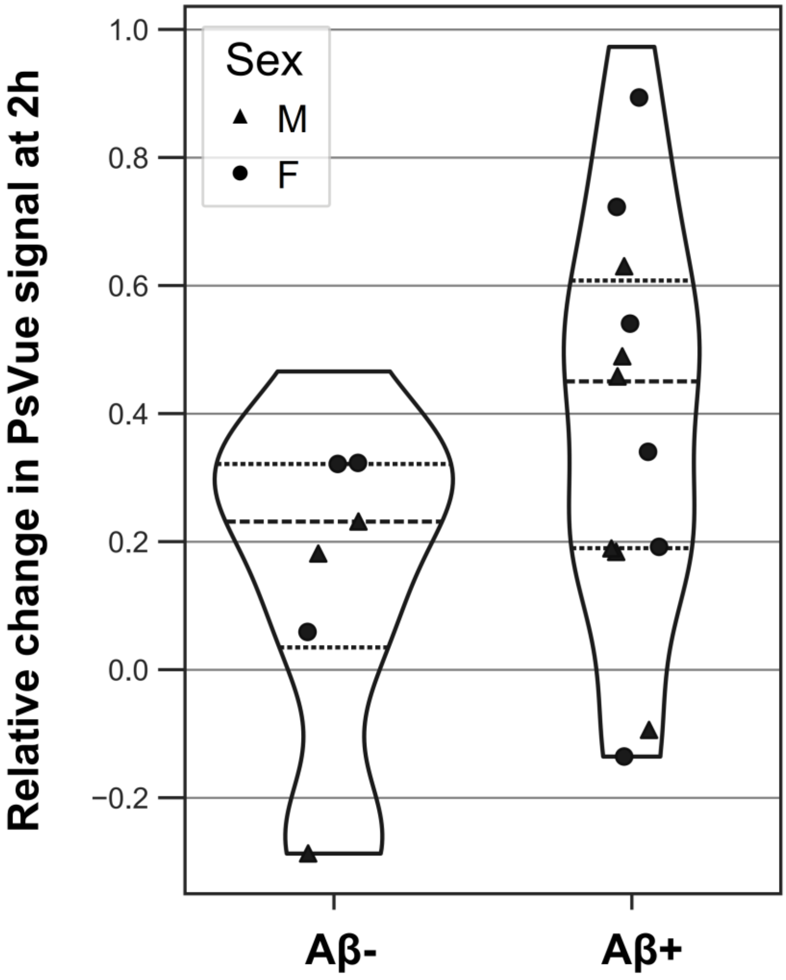

Figure S10 Increased exposure of phosphatidyl serine after BH challenge.
ΔPsVue/PsVue change for individual fields of view after 2h treatment with Aβ+ or Aβ- brain homogenate.

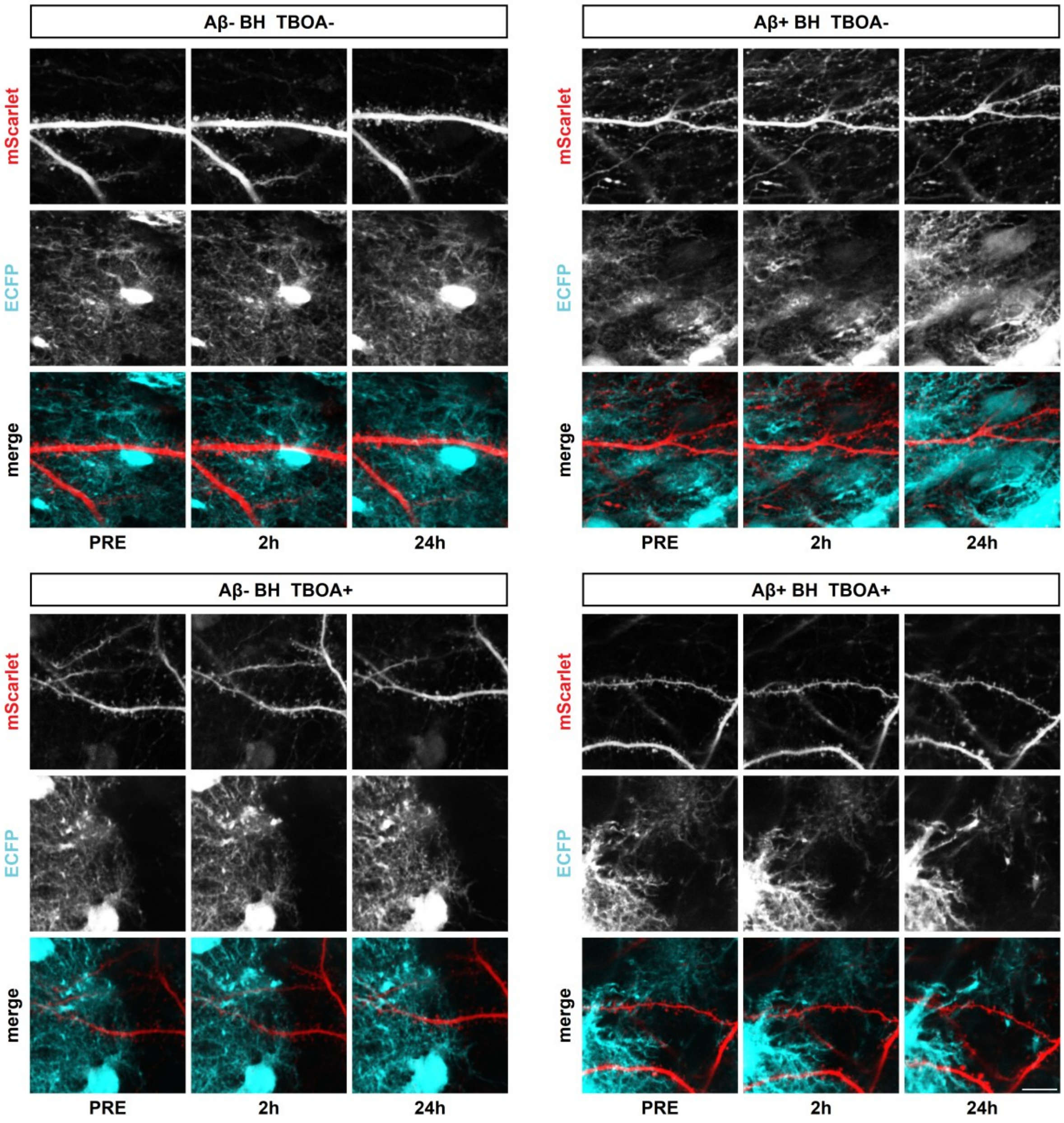

Figure S11 Full panels of images cropped in Figure 5.

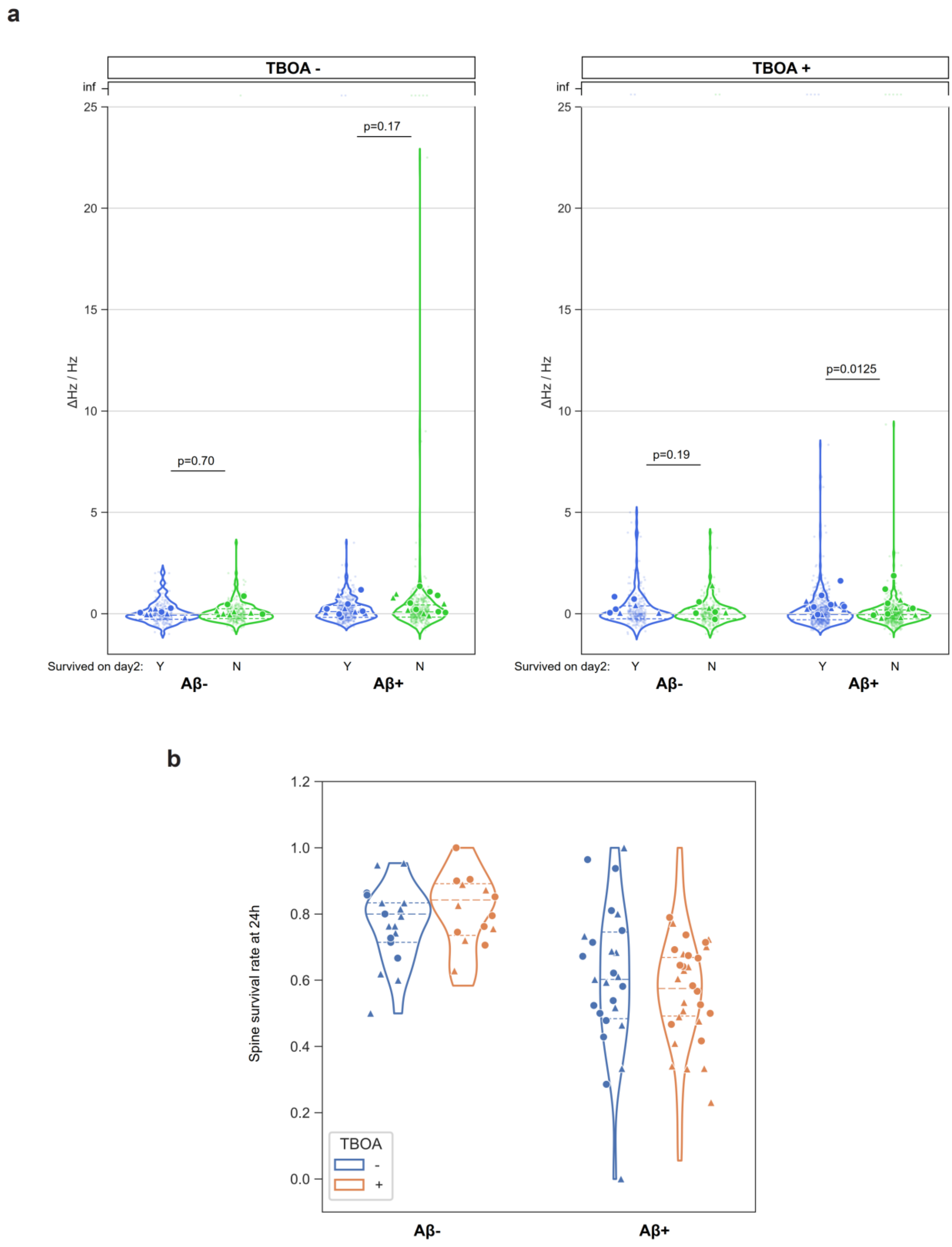

Figure S12 Changes in activity by proximity of astrocytes. a) ΔHz/Hz for synapses treated with Aβ- or Aβ+ BH, with either vehicle or TBOA. c) Overall synapse survival at 24h after treatment with Aβ- or Aβ+ BH, with either vehicle or TBOA
